# *Streptococcus mutans* Displays Altered Stress Responses While Enhancing Biofilm Formation by *Lactobacillus casei* in Mixed-Species Consortium

**DOI:** 10.3389/fcimb.2017.00524

**Published:** 2017-12-20

**Authors:** Zezhang T. Wen, Sumei Liao, Jacob P. Bitoun, Arpan De, Ashton Jorgensen, Shihai Feng, Xiaoming Xu, Patrick S. G. Chain, Page W. Caufield, Hyun Koo, Yihong Li

**Affiliations:** ^1^Center of Oral and Craniofacial Biology, Louisiana State University Health Sciences Center, New Orleans, LA, United States; ^2^Department of Comprehensive Dentistry and Biomaterials, Louisiana State University Health Sciences Center, New Orleans, LA, United States; ^3^Department of Microbiology, Immunology and Parasitology, Louisiana State University Health Sciences Center, New Orleans, LA, United States; ^4^Genome Science Group, Bioscience Division, Los Alamos National Laboratory, Los Alamos, NM, United States; ^5^Basic Science and Craniofacial Biology, New York University College of Dentistry, New York, NY, United States; ^6^Biofilm Research Labs, Levy Center for Oral Health, Department of Orthodontics, School of Dental Medicine, University of Pennsylvania, Philadelphia, PA, United States

**Keywords:** *S. mutans*, oral lactobacilli, mixed-species biofilms, dental caries, RNA-seq

## Abstract

Like *Streptococcus mutans*, lactobacilli are commonly isolated from carious sites, although their exact role in caries development remains unclear. This study used mixed-species models to analyze biofilm formation by major groups of oral lactobacilli, including *L. casei, L. fermentum, L. rhamnosus, L. salivarius* ssp. *salivarius*, and *L. gasseri*. The results showed that lactobacilli did not form good biofilms when grown alone, although differences existed between different species. When grown together with *S. mutans*, biofilm formation by *L. gasseri* and *L. rhamnosus* was increased by 2-log (*P* < 0.001), while biofilms by *L. fermentum* reduced by >1-log (*P* < 0.001). *L. casei* enhanced biofilm formation by ~2-log when grown with *S. mutans* wild-type, but no such effects were observed with *S. mutans* deficient of glucosyltransferase GtfB and adhesin P1. Both *S. mutans* and *L. casei* in dual-species enhanced resistance to acid killing with increases of survival rate by >1-log (*P* < 0.001), but drastically reduced the survival rates following exposure to hydrogen peroxide (*P* < 0.001), as compared to the respective mono-species cultures. When analyzed by RNA-seq, more than 134 genes were identified in *S. mutans* in dual-species with *L. casei* as either up- or down-regulated when compared to those grown alone. The up-regulated genes include those for superoxide dismutase, NADH oxidase, and members of the mutanobactin biosynthesis cluster. Among the down-regulated genes were those for GtfB and alternative sigma factor SigX. These results further suggest that interactions between *S. mutans* and oral lactobacilli are species-specific and may have significant impact on cariogenic potential of the community.

## Introduction

The oral microbiome, represented by dental plaque, harbors diverse and abundant microbial communities consisting of over 700 different species or phylotypes (Jenkinson, 2011). Both intra- and inter-species interactions in the oral flora have been well documented, although the underlying mechanisms remain unclear (Kuramitsu et al., 2007). The oral cavity is featured with fluctuating and often unpredictable conditions, such as nutrient source and availability and pH. Both microbe-microbe and microbe-environment interactions can profoundly influence the composition and relative proportions of major groups in the dynamic communities, leading to dysbiosis and consequently, development of oral diseases such as dental caries and periodontitis (Kuramitsu et al., 2007; Jenkinson, 2011; Burne et al., 2012; Hajishengallis et al., 2017). Cariogenic plaque, for instance, is characterized by dramatic increases in the proportion of acidogenic and aciduric species, which include mutans streptococci and lactobacilli (Jenkinson, 2011).

As a major causative agent of dental caries, *S. mutans* possesses multiple mechanisms to colonize and persist on the tooth surface, and under certain conditions to become numerically significant, causing carious lesions (Hamada and Slade, 1980; Bowen et al., 1991; Bowen and Koo, 2011; Burne et al., 2011). Multi-functional adhesin P1 (also Antigen I/II, SpaP, or PAc) functions as the primary factor mediating early attachment to the tooth surface via interaction with salivary agglutinin-gp340 (Crowley et al., 1993). *S. mutans* also produces at least three glucosyltransferases (GtfB, -C, and -D) that polymerize the glucosyl moiety from sucrose, generating adhesive glucans (Bowen and Koo, 2011). The Gtfs and their glucan products, along with the glucan-binding proteins (Gbps), constitute the sucrose-dependent pathway central in plaque formation and caries development (Banas et al., 2007; Gregoire et al., 2011). In addition, multiple two-component signal transduction systems, molecular chaperones, and biofilm regulatory protein BrpA are shown to play an important role in *S. mutans* biofilm formation (Burne et al., 2011).

As the first microorganisms implicated in human dental caries (Owen, 1949), lactobacilli are frequently identified at carious sites, esp. in patients with advanced caries, with *L. casei, L. fermentum, L. gasseri, L. salivarius*, and *L. rhamnosus* among the most prevalent groups (Caufield et al., 2007; Badet and Thebaud, 2008; Gross et al., 2012). Lactobacilli can utilize various kinds of sugars, generating lactic acid and other organic acids, and are highly resistant to low pH. Previously, our *in vitro* model studies showed that *L. casei* alone did not colonize a surface efficiently, but drastically increased its ability to colonize and accumulate biofilms during growth with *S. mutans* (Wen et al., 2010). Similar results were also observed with *Actinomyces* spp. (Van Houte et al., 1991; Filoche et al., 2004; Wen et al., 2010). Our previous studies have shown that *S. mutans* in dual-species cultures with several other prominent bacterial species including *L. casei* had altered expression of several genes known to be critical to biofilm formation and cariogenicity (Wen et al., 2010). In this study, representatives of lactobacilli groups most frequently isolated from the carious sites were first analyzed using *in vitro* mixed-species models for their abilities to form biofilms with and without the presence of *S. mutans*. An *in vitro* continuous biofilm model, transcriptional profiling via RNA-seq and metabolite analysis via HPLC were then used to further investigate the interactions between *S. mutans* and *L. casei* and identify the factors that mediate these interspecies interactions. Results demonstrated that interactions between *S. mutans* and the major oral lactobacilli are species-specific and may have an impact on the pathogenicity of the community.

## Materials and methods

### Bacterial strains and cultivation

All bacterial strains used in this study are listed in Table 1. *S. mutans* strains were maintained in Brain Heart Infusion (BHI, Becton, Dickinson and Company, Sparks, MD). Lactobacilli were maintained in Lactobacillus MRS (Difco Laboratories, MI). For *Veillonella dispar*, tryptic soy broth (TSB, Becton, Dickinson and Company, Sparks, MD) plus lactic acid at 0.6% (v/v) was used (Liu et al., 2011). For *S. mutans* and lactobacilli dual-species biofilm formation, semi-defined biofilm medium (BM) with 18 mM glucose and 2 mM sucrose (BMGS) was used (Loo et al., 2000; Bitoun et al., 2012). For triple-species biofilms, *S. mutans*, lactobacilli and *V. dispar* were grown in TSB. For transcriptional profiling and metabolite analysis, chemically defined medium FMC was used with modifications (mFMC) in an effort to facilitate the growth of lactobacilli (Terleckyj et al., 1975; Elli et al., 2000; Savijoki et al., 2006; Wegkamp et al., 2009; Table S1). All solid media were prepared similarly with inclusion of Bacto agar (Difco Laboratories, Franklin Lakes, NJ) at the level of 1.5% (w/v). When needed, erythromycin (5 μg/ml), and/or kanamycin (1 mg/ml) were added to the proper medium. Unless otherwise stated, bacterial cells were grown at 37°C in an aerobic chamber with 5% CO_2_.

**Table 1:** Bacterial strains used in this study.

**Strains /Plasmid**	**Relevant characteristics**	**References/Source**
*S. mutans* UA159	wild-type	Ajdic et al., 2002
*S. mutans* SAN136	*ΔspaP*, Kan^r^	Ahn et al., 2008
*S. mutans* SAB106	*ΔgtfB*, Kan^r^	The Burne lab
*S. mutans* SAB108	*ΔgtfC*, Kan^r^	The Burne lab
*S. mutans* SAB109	*ΔgtfBC*, Kan^r^	The Burne lab
*S. mutans* SAB110	*Δftf*, Kan^r^	The Burne lab
*S. milleri* KSB8	*gtfB of S. mutans* GS5, Em^r^	Vacca-Smith and Bowen, 1998
*L. casei* 4646	wild-type	Shu et al., 2003
*L. casei* CB	derivative of 4646, Erm^r^	Wen et al., 2010
*L. fermentum* ATCC 14931	wild-type	ATCC^#^
*L. rhamnosus* ATCC7469	wild-type	ATCC^#^
*L. salivarius* ssp. *salivarius* ATCC 11741	wild-type	Caufield et al., 2015
*L. gasseri* ATCC 33323	Wild-type	Caufield et al., 2015
*L. gasseri* M57-23	mother-caries active^*^	Caufield et al., 2015
*L. fermentum* M87-28	mother-caries active^*^	Caufield et al., 2015
*L. salivarius* M87-8	mother-caries active^*^	Caufield et al., 2015
*L. rhamnosus* M72-26	mother-caries active^*^	Caufield et al., 2015
*V. dispar* ATCC17745	wild-type	Shu et al., 2003

*Erm^r^ and Kan^r^ for erythromycin and kanamycin resistance, respectively*.

# *strains purchased directly from American Type Culture Collection, Manassas, VA*;

* *Clinical strains previously isolated from mothers of caries active kids*.

### Growth of mixed-species biofilms

For biofilm formation, 96-well plates and glass slides were used as substratum, and biofilms were grown by following protocols described previously (Loo et al., 2000; Wen and Burne, 2002; Wen et al., 2010). For the 96-well plate model, actively growing individual species were diluted by 1:100 (v/v) in proper growth medium, and 200 μL aliquots were transferred to the wells of the culture plates (Corning, New York) in triplicate. For dual- and triple-species biofilms, individual species were mixed proportionally and then diluted as described above. The plates were incubated at 37°C in an aerobic chamber with 5% CO_2_ or for biofilms involving veillonella, in an anaerobic box for 24 and 48 h. By the end of incubation, the adherent biofilms were stained with 0.1% crystal violet and quantified using a spectrophotometer at 575 nm (Loo et al., 2000; Wen and Burne, 2002; Wen et al., 2010). For the glass slides model, the biofilms were grown on the glass slides in 50 ml Falcon tubes, and the slides were aseptically transferred daily to fresh medium (Loo et al., 2000; Wen and Burne, 2002; Wen et al., 2010). For enumeration of the different species, aliquots of the mixed-species cultures were plated on BHI agar, where the two bacteria form colonies with distinctive morphological characteristics, and on Rogosa agar (Becton, Dickinson and Company, Sparks, MD), a selective medium for lactobacilli. For continuous flowing conditions, *S. mutans* and *L. casei* were grown in mFMC with glucose (18 mM) and sucrose (2 mM), and biofilms were grown on glass slides deposited in a Drip Flow Biofilm Reactor (BioSurface Technologies, Montana). The system was run with a medium flow rate of 60 ml per hour in a 37°C warm room for 48 and 120 h, respectively (Wen et al., 2010; Fan et al., 2012). By the end of the experiments, the biofilms were scratched off with a sterile spatula and suspended in 5 ml potassium phosphate buffer, 10 mM, pH 7.0, sonicated briefly to de-chain and separate the cells (Wen et al., 2010), and serial dilutions were then plated in triplicate on proper agar medium for enumeration of viable biofilm cells (Fan et al., 2012).

### GtfB binding to *L. casei* surface and fluorescence imaging of glucan production on *L. casei* and *S. mutans* bound to *L. casei*

The GtfB enzyme was prepared from culture supernatant of *S. milleri* KSB8 harboring *S. mutans gtfB* and purified via hydroxyapatite column chromatography using our well-established protocol as detailed previously (Vacca-Smith and Bowen, 1998; Gregoire et al., 2011). The *in vitro* Gtf binding assay and fluorescence imaging were carried out similarly as described by Gregoire et al. (2011). Briefly, for *in vitro* binding assay, *L. casei* cells (~1 × 10^9^ cfu/ml) were mixed with saturating amount of GtfB (25 μg/ml, 3 U), in adsorption buffer and incubated for 60 min at 37°C. For controls, *L. casei* cells were incubated in adsorption buffer alone. Following adsorption, the cells were stained with nucleic acid staining dye SYTO 9 (485/498 nm; Molecular Probes, Eugene, OR) that confers green fluorescence. Then, the cells were washed twice with adsorption buffer by centrifugation, and the pellets were resuspended in adsorption buffer. For glucan production, 1 μM Alexa Fluor 647-conjugated dextran (647/688 nm, Molecular Probes) was added to the reaction mixture containing *L. casei* (1 × 10^9^ cells/ml) with and without surface-adsorbed GtfB and with and without sucrose (100 mM) for 1 h at 37°C. Following incubation, 20 μl of the mixture were placed on a glass slide and imaging was performed using an upright Olympus confocal microscope (Olympus Fluoview BX61). For *L. casei-S. mutans* interactions, *S. mutans* cells were washed with adsorption buffer similarly as described above, and stained with DAPI (4′,6-diamidino-2-phenylindole) blue fluorescence nucleic acid staining dye (358/461 nm; Molecular Probes). The fluorescently labeled cells (1 × 10^9^ cells/ml) were mixed with *L. casei* with and without bound GtfB and *L. casei* with and without surface glucan coating, and incubated at 37°C for 1 h. After incubation, the suspension was immediately visualized under confocal microscope.

### Acid and hydrogen peroxide killing assays

To evaluate the impact of growth in a consortium on stress tolerance response, the abilities of *S. mutans* and *L. casei* to withstand low pH and oxidative stress were evaluated using acid and hydrogen peroxide killing assays (Wen and Burne, 2004; Wen et al., 2006, 2010). For acid killing assays, 48-h biofilms of *S. mutans* and *L. casei* mono- and dual-species biofilms were grown on glass slides in BMGS. Biofilms were briefly sonicated to detach and de-chain *S. mutans*, washed once, and the acid killing assay was carried out by incubation of the bacterial cells in glycine buffer of pH 2.0, 0.1 M for *L. casei* and pH 2.8, 0.1 M for *S. mutans* for a period of 15, 30, 45 and 60 min (Wen and Burne, 2004; Wen et al., 2006, 2010). For hydrogen peroxide killing assay, the mono- and dual-species cultures were grown in BHI broth until early-exponential phase (OD_600nm_ ≅0.4), and washed once as described above before subjecting to the killing assays by incubation of the bacterial cells in glycine buffer containing 0.2% hydrogen peroxide for period of 60, 90, 105, and 120 min as described elsewhere (Wen and Burne, 2004; Wen et al., 2006, 2010).

### Competition assays on agar plates

Competitions between *S. mutans* and the lactobacilli were examined by following the method of Kreth et al. (2005).

### Organic acid analysis

Organic acids were determined using UV-HPLC (Kara et al., 2006 88; Ausubel et al., 2010). For acidic metabolite analysis, mono- and dual species biofilms of *S. mutans* and *L. casei* were grown on glass slides in mFMC with 18 mM glucose and 2 mM sucrose for 24- and 48-h. At the end of incubation, the cultures were spun down, and supernates were collected and filtered via centrifugal units (Millipore, 10kDa MWCT). The supernates were then diluted 10-fold in 40 mM Na_2_SO_4_, pH 2.65, and passed over a Acclaim™ OA 3 μm column (DX070087, Dionex) optimized for organic acids, at 0.21 mL/min of 40 mM Na_2_SO_4_ mobile phase at 30°C on a Agilent 1100 Series UV/HPLC. Samples were detected at 210 nm. Sodium salts of fumaric, α-ketoglutaric, citric, proponic, butyric, succinic, and lactic acid (Sigma) were used to prepare single and mixed standard solutions in deionized water.

### Transcriptomic analysis via RNA-Seq

For RNA extraction, early-exponential phase cultures, OD_600nm_ ~0.3, were treated with RNAProtect (Qiagen Inc., CA), and total RNAs were extracted using a hot phenol method as detailed elsewhere (Wen and Burne, 2004; Wen et al., 2006). To remove all DNA, the purified RNAs were treated with DNase I (Ambion, Inc., TX) and RNA was retrieved with RNeasy Mini kit (Qiagen, Inc.), including an additional on-column DNase I treatment with RNase-free DNase I. The RNA samples were depleted of ribosomal RNA using Illumina's Ribo-Zero Bacterial Kit. 40 ng of RNA were then converted to cDNA, and libraries were generated using Illumina's ScriptSeq v2 Library Preparation Kit. Deep sequencing and post-sequencing analysis were carried out by following our established protocols (Robinson et al., 2010; Lo and Chain, 2014; Love et al., 2014; Maekawa et al., 2014; Kim et al., 2015).

### Statistical analysis

Unless specified otherwise, all quantitative data were further analyzed by student's *t*-test or ANOVA and Tukey's pairwise comparison. A difference of *P* < 0.05 is considered as statistically significant.

## Results

### Lactobacilli differ in the ability to form biofilms

**Figure 1:**
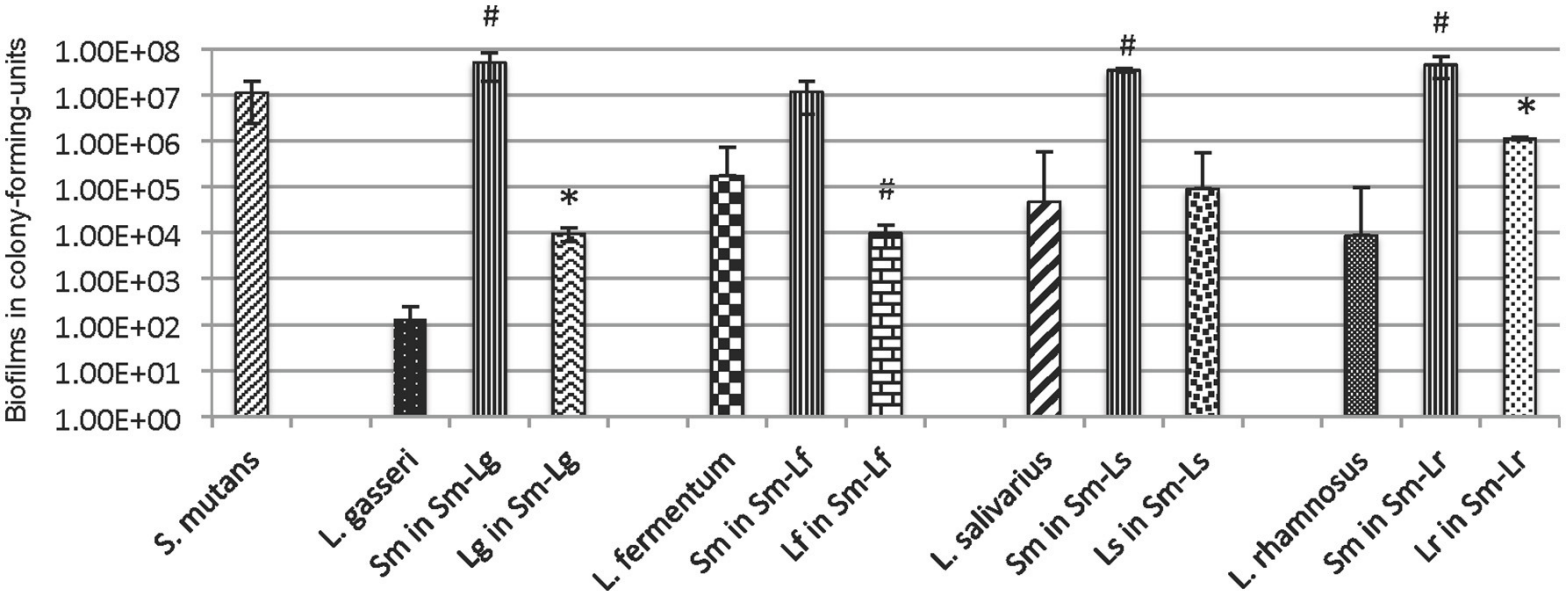
Forty eight-hour biofilms of *L. gasseri* (Lg), *L. fermentum* (Lf), *L. salivarius* (Ls) and *L. rhamnosus* (Lr) in mono-species and dual-species with *S. mutans* (Sm) grown in BM plus glucose (18 mM) and sucrose (2 mM) on glass slides vertically deposited in 50 ml Falcon tubes. Data represent the average (±standard deviation in error bars) of at least three separate experiments, with ^*^ and # indicating statistical difference at *P* < 0.001 and 0.05, respectively, when compared to the respective mono-species biofilms.

Five different species of lactobacilli that are most frequently isolated from carious sites were selected for the study (Table 1; Caufield et al., 2007, 2015; Badet and Thebaud, 2008; Gross et al., 2012). Like *L. casei* (Van Houte et al., 1972; Wen et al., 2010), all lactobacillus strains showed only limited abilities to form biofilms on glass, polystyrene and hydroxylapatite surfaces tested. When grown in BMGS, *L. fermentum* formed the best biofilms when grown alone over night, followed by *L. salivarius* ssp. *salivarius*, and *L. rhamnosus*, while *L. gasseri* possessed the least capacity to form biofilms under the conditions studied (Figure 1). When compared between 24- and 48-h incubation (without growth medium refreshment), 48-h incubation significantly reduced biofilm formation for all lactobacilli tested (Figure S1). When grown in TSB in 96-well plates, *L. fermentum* again accumulated the most biofilms overnight among the lactobacilli tested, while the least biofilms were observed with *L. rhamnosus* (Figure S2). Continuous incubation in TSB for 48 h moderately increased biofilms by *L. casei* and *L. salivarius* ssp. *salivarius*, while biofilms by *L. gasseri* and *L. fermentum* were reduced moderately. Interestingly, when grown in TSB on glass slides for 5 days, the highest biofilm was observed with *L. rhamnosus*, averaging just 8.7E7 ± 7.1E6 in colony-forming-units (CFU) (Figure S3). It was followed by *L. fermentum*, and *L. salivarius* ssp. *salivarius*, and the least was obtained with *L. gasseri*, with an average of 3.3E4 ± 4.4E3 in CFU. Selected clinical isolates of different major species, which were isolated from mothers of caries active kids from previous studies (Caufield et al., 2015), were also analyzed. Similar trends were also observed when they were grown in TSB, with the most biofilms produced by *L. fermentum*, followed by *L. salivarius, L. gasseri*, and the least was found with *L. rhamnosus* (Data not shown).

### Interactions between *S. mutans* and lactobacilli are species-specific

When compared to mono-species cultures grown in BMGS, *L. gasseri* and *L. rhamnosus* increased biofilm formation by ~2-log after 48 h when grown together with *S. mutans* (*P* < 0.001) (Figure 1, Figure S1). In contrast, biofilms of *L. fermentum* decreased by >1-log during growth with *S. mutans* (*P* < 0.05). No significant differences were measured with *L. salivarius* ssp. *salivarius* in dual-species with *S. mutans* in comparison to those grown alone. On the other hand, some significant increases were also observed with *S. mutans* when grown together with *L. gasseri, L. salivarius* ssp. *salivarius*, and *L. rhamnosus* (*P* < 0.05), while moderate decreases were measured after 24 h' growth with *L. fermentum* (Figure S1). When grown in a consortium in TSB in 96 well plates, the amount of total biofilms was decreased significantly when *S. mutans* was partnered with *L. fermentum, L. salivarius* ssp. *salivarius*, and *L. rhamnosus*, while moderate increases of biofilms were observed with *S. mutans-L. gasseri* biofilms (Figure S2). On glass slides (Figure S3), co-cultivation with *S. mutans* for 5 days significantly increased biofilms of *L. salivarius* ssp. *salivarius* (*P* < 0.05), while significantly reduced biofilms of *L. fermentum* (*P* < 0.05) and *L. gasseri* (*P* < 0.001). This is contrary to what were seen with 24- and 48-h biofilms in BMGS, when compared to the respective mono-species counter-parts. No significant differences were measured between *S. mutans* grown alone and those co-cultivated with the lactobacilli tested under these conditions (Figure S3).

### GtfB and P1 play an important role in mixed-species biofilm formation

**Figure 2:**
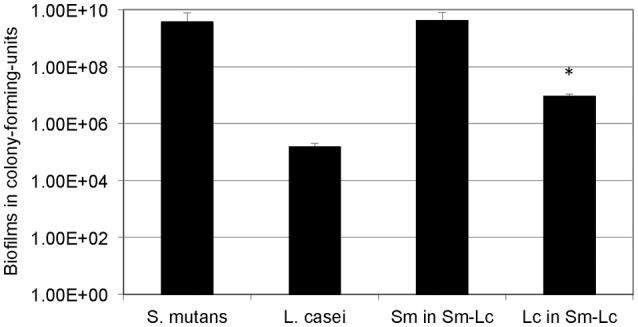
Five-day biofilm grown under continuous flowing conditions. *S. mutans* (Sm) and *L. casei* (Lc) mono- and dual-species biofilms were grown on glass slides deposited in a Drip Flow Biofilm Reactor in mFMC containing 18 mM glucose and 2 mM sucrose. Results represent the average (±standard deviation in error bars) of at least three separate experiments, with ^*^ indicating statistical difference at *P* < 0.001 relative to its mono-species biofilms.

**Figure 3:**
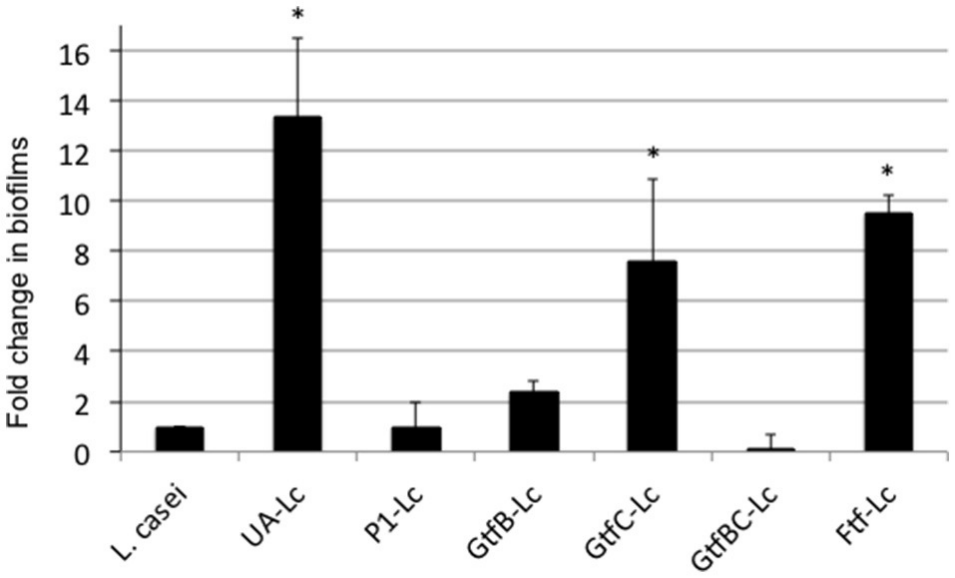
*L. casei* biofilm formation with *S. mutans* mutants. *L. casei* (Lc) was grown alone or together with *S. mutans* wild-type UA159 (UA) and its mutants deficient of P1, GtfB, GtfC, GtfBC, or Ftf in biofilm medium with glucose and sucrose for 48-h. Data are expressed as the ratio of *L. casei* biofilms (in colony-forming-units) in dual-species over those in mono-species, with ^*^ indicating significant differences at *P* < 0.001.

When grown in flow cells in mFMC for 5 days, *L. casei* alone accumulated a little over 1.3E5 ± 5.5E4 CFU per mL, but when grown together with *S. mutans*, its biofilm increased by 54-fold, averaging 7.0E6 ± 1.9E5 CFU (*P* < 0.001; Figure 2). No significant differences were observed between *S. mutans* grown alone and those in dual-species with *L. casei*. In an effort to find out if Gtf enzymes were involved in mixed-species biofilm formation, *L. casei* was grown in BMGS with *S. mutans* strains deficient of GtfB, GtfC, GtfB&C, Ftf (for fructosyltransferase, Ftf), and P1. The *gtf* and *ftf* mutants were generated by standard allelic replacement strategies and kindly provided by Robert A. Burne (Department of Oral Biology, University of Florida, Gainesville, FL). Consistently, co-cultivation of *L. casei* with the wild-type *S. mutans* UA159 increased biofilm formation by *L. casei* by >13-fold (*P* < 0.001), when compared to *L. casei* grown alone (Figure 3). Relatively, biofilm formation by *L. casei* increased by 8-fold when grown with a GtfC-deficient mutant (*P* < 0.001), by >9-fold (*P* < 0.001) when co-cultivated with an *ftf-*deficient mutant, but no significant differences were observed when incubated with GtfB- and GtfBC- and P1-deficient mutants (*P* > 0.05).

**Figure 4:**
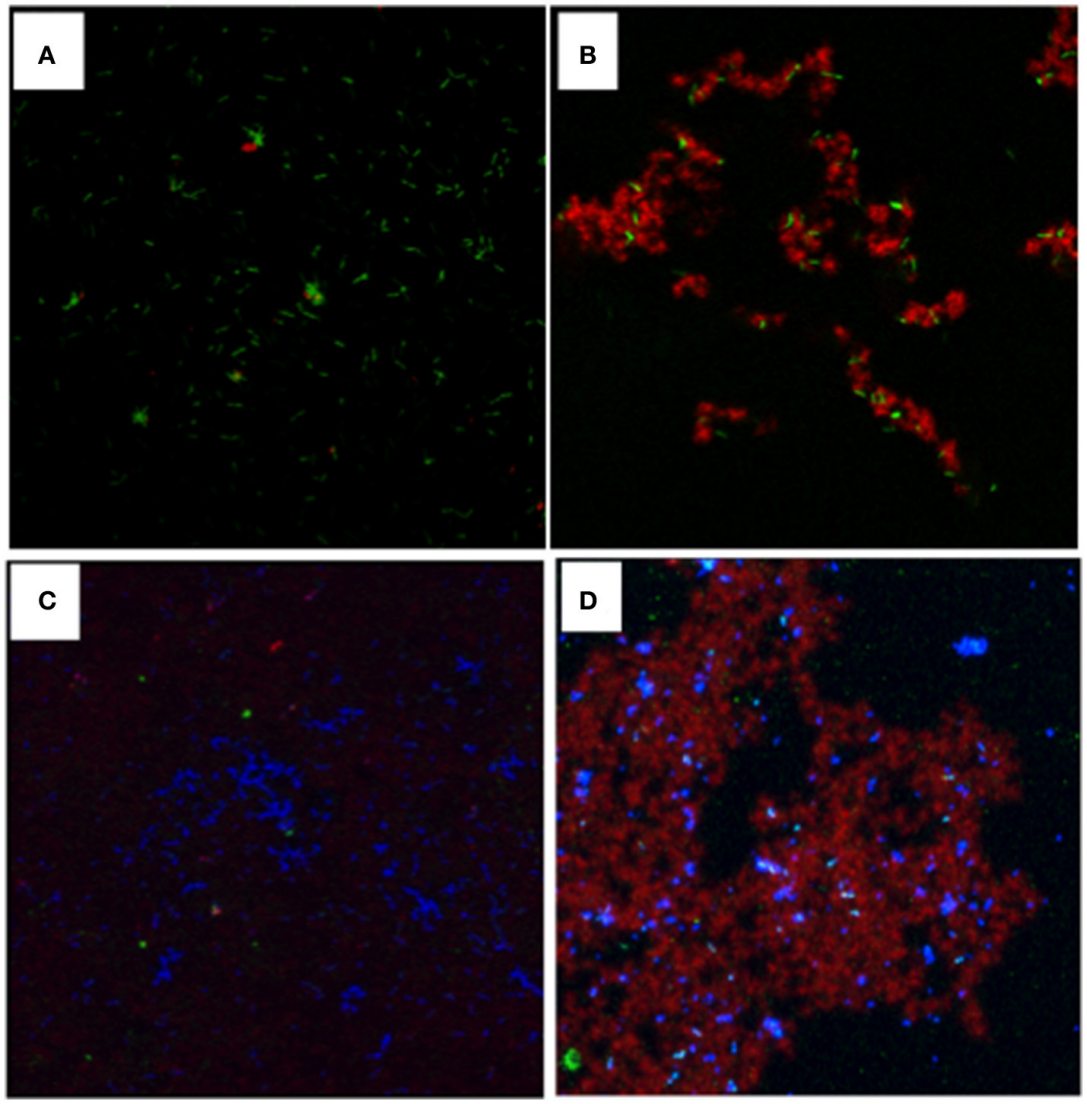
Visualization of glucans synthesized *in situ* by GtfB adsorbed on *L. casei* and *S. mutans* binding to *L. casei* with and without glucans. For glucan visualization, *L. casei* was incubated with GtfB or buffer, and following washes, exposed to sucrose for 1 h, and glucans were imaged using a confocal microscope. **(A)** *L. casei* cells (in green) with GtfB incubated in buffer alone; **(B)** *L. casei* with bound-GtfB incubated with sucrose showing rich glucans (in red) engulfed in green *L. casei* cells. For interactions, *S. mutans* were mixed proportionally with *L. casei* with and without GtfB glucan-coating. **(C)** *S. mutans* (in blue) incubated with green *L. casei* with no glucans on surface; and **(D)** *S. mutans* (in blue) incubated with *L. casei* coated with GtfB glucans (in red). Images were obtained using an upright Olympus confocal microscope with a 100x oil objective.

When analyzed using *in vitro* assays, the purified GtfB from *S. mutans* (Gregoire et al., 2011) was shown to readily bind to *L. casei* cells, and the bound GtfB was able to generate glucose polymers in the presence of sucrose (Figures 4A,B). When mixed together, *S. mutans* was shown to be able to bind to *L. casei*, forming microcolony-like structures with green *L. casei* (Data not shown). In contrast, more *S. mutans* cells were found on *L. casei* with GtfB-bound (data not shown) and especially, on the top of the glucan matrices surrounding the *L. casei* cells (Figures 4C,D), as compared to the controls with no GtfB glucans.

### *V. dispar* and *S. mutans* enhance biofilm formation when grown together

*V. dispar* did not form good biofilm when grown in TSB alone (Figure S2). When it was grown with *S. mutans*, the amount of mixed-species biofilms was significantly increased, especially for those grown continuously for 48 h (Figure S2). However, no such effects were observed when *V. dispar* was co-cultivated with the different species of lactobacilli tested. When *S. mutans* and *V. dispar* were grown together with different lactobacilli, the amounts of mixed-species biofilms were very similar to what were measured with the *S. mutans-V. dispar* dual-species biofilms, regardless of the lactobacilli in the consortium (Figure S2).

### *S. mutans* and *L. casei* in consortium enhanced acid tolerance responses and reduced survival rates against hydrogen peroxide killing

**Figure 5:**
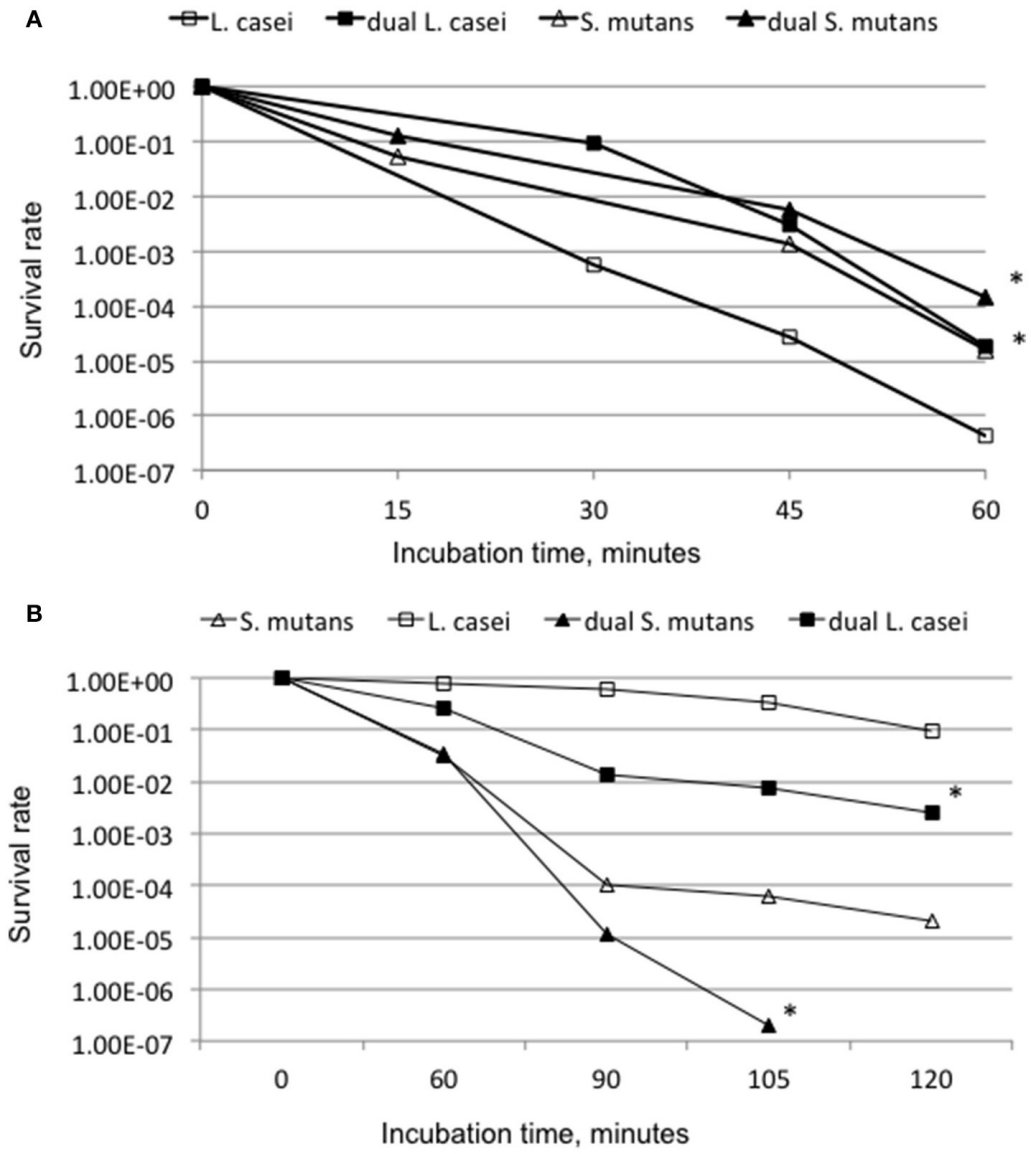
Acid **(A)** and hydrogen peroxide **(B)** killing assays. **(A)** For acid killing, *S. mutans*, and *L. casei* biofilms were grown in biofilm medium with glucose and sucrose on glass slides individually or in dual-species for 48 h, and acid killing was carried out at pH 2.0 for *L. casei* and 2.8 for *S. mutans*. **(B)** For hydrogen peroxide killing assays planktonic cultures of *S. mutans* and *L. casei* were grown overnight in BHI broth, and killing assays were done in glycine buffer containing 0.2% hydrogen peroxide. Data presented here are representatives of more than three independent assays with ^*^ indicating significant difference at *P* < 0.001 in comparison to the respective mono-species cultures.

When compared to the respective mono-species cultures, both *S. mutans* and *L. casei* grown in dual-species consortium increased their survival rate by more than 1-log (*P* < 0.001) after incubation at pH 2.0 for *L. casei* and 2.8 for *S. mutans* for a period of 60 min (Figure 5A). When analyzed by hydrogen peroxide killing assay, *S. mutans* grown in dual-species reduced its survival rate by >1-log after 90 min, >2-log after 105 min and became undetectable after 120 min, as compared to those grown individually (*P* < 0.001; Figure 5B). Relatively, *L. casei* displayed higher resistance to hydrogen peroxide than *S. mutans*, although it also had a reduction of survival rate by >1-log for cells grown together with *S. mutans* (*P* < 0.05).

### *S. mutans* produces antimicrobials against *L. fermentum*

*Streptococcus mutans* is known to produce mutacins that possess a broad spectrum of activities against closely associated bacterial species (Merritt and Qi, 2012). Several oral *Lactobacillus* spp. have also been shown to produce bacteriocins effective against *S. mutans* (Simark-Mattsson et al., 2007; Strahinic et al., 2007; Wu et al., 2015). When analyzed using a competition assay, *S. mutans* displayed inhibitory activity against *L. fermentum* when allowed to grow first on half-strength BHI agar plates. This was illustrated by a crescent zone of inhibition, but no such activity was detected when *S. mutans* and *L. fermentum* were spotted next to each other simultaneously nor was when *L. fermentum* was allowed to grow first (Figure S4). *L. fermentum* did not produce any visible inhibitory activity under the conditions studied. No apparent inhibitory activities were observed when the two bacteria were grown on regular BHI agar plates. No antimicrobial activity was detected from *S. mutans* against the other lactobacilli tested, or vice versa.

### *S. mutans* and *L. casei* in dual-species had different metabolic profiles from those grown individually

For metabolite analysis, a chemically defined medium, termed mFMC, was developed using FMC as the basal medium and with defined lactobacillus medium as a reference (Table S1). This medium retains its strong support for *S. mutans* of FMC, while enhancing the growth of lactobacilli (Figure 2). When the dual-species overnight cultures of *S. mutans* and lactobacilli tested were grown in mFMC with 18 mM glucose and 2 mM sucrose and analyzed using a micro pH probe, all except *L. fermentum*, had a reduced cultural pH than the respective mono-species cultures (data not shown). Continuous incubation for 48 h led to pH elevation for both *S. mutans* and the lactobacilli strains, but again, the mixed-species cultures, including those with *L. fermentum*, had a significantly reduced pH than when they grew alone. When the organic acid profile was analyzed using HPLC, succinic acid was reduced by 4-fold in 24-h dual-species biofilms, as compared to the respective *S. mutans* and *L. casei* mono-species cultures (Figure S5). Other more subtle changes were also identified for lactic acid, α-ketoglutaric acid, fumaric acid, and citric acid. In addition, metabolites with major alterations with retention times of ~4 and ~8 min were also apparent under the conditions studied, although the exact identity of these metabolites currently remain unknown.

### *S. mutans* in dual-species has an altered transcriptional profile

When analyzed by deep- sequencing, the transcriptional profile of *S. mutans* in dual-species with *L. casei* was shown to be significantly different from that in mono-species with >134 genes being identified as either up- or down regulated (*P* < 0.001; Table S2). Of the altered genes, six were down-regulated by more than 1.5-fold, and twelve were up-regulated by >1.5-fold. Among the down-regulated genes were those for GtfB, dextranase, and alternative sigma factor (SigX) (Table 2). The up-regulated genes include those for GtfD, Ftf, H_2_O-forming NADH oxidase (Nox1), Mn-dependent superoxide dismutase (SodA), lipid hydroperoxide peroxidase (Tpx), and members of the gene cluster for biosynthesis of mutanobactin (Wu et al., 2010).

**Table 2:** Selected genes identified by RNA-Seq analysis.

**Locus**	Description^*^	**Ratio (D/M)^#^**	***P*-value**
SMU.265	Putative amino acid kinase	1.56	0.016654713
SMU.270	Unknown	1.67	0.000835434
SMU.629	Mn-dependent Superoxide dismutase, SodA,	1.60	2.48242E-08
SMU.910	Glucosyltransferase-S, GtfD	1.50	1.98036E-14
SMU.924	Lipid hydroperoxide peroxidase, Tpx	1.67	1.5412E-12
SMU.1117	NADH oxidase (H_2_O-forming), Nox1, Nox	2.09	5.75842E-26
SMU.1339	Putative bacitracin synthetase	1.65	6.9306E-08
SMU.1340	Putative surfactin synthetase	1.72	2.61792E-10
SMU.1342	Putative bacitracin synthetase, BacA	1.92	5.72903E-15
SMU.1346	Putative thioesterase, BacT	2.15	2.98087E-12
SMU.1425	Putative Clp proteinase, ATP-binding subunit, ClpB	1.53	0.008209022
SMU.2028	Levansucrase precursor, beta-D-fructosyltransferase, Ftf, SacB	1.72	7.67161E-26
SMU.20	Unknown (MreC, putative cell shape determining protein)	−1.61	2.98587E-05
SMU.183	Putative Mn/Zn ABC transporter	−1.65	0.011069851
SMU.184	Putative ABC transporter metal binding lipoprotein	−1.69	7.99849E-07
SMU.1004	Gucosyltransferase-I, GtfB	−1.50	1.11398E-16
SMU.1997	Transcriptional regulator ComX, alternative sigma factor	−1.59	9.38051E-05
SMU.2042	Dextranase precursor	−1.66	3.37282E-19

* *Description and putative function of the identified genes are based on the published S. mutans database*.

# *Defined as relative levels of expression in the dual-species over those in the mono-species with “–” indicating the down-regulated genes*.

## Discussions

While lactobacilli are frequently isolated from carious sites (Van Houte et al., 1972; Michalek et al., 1981), this study has generated evidence that none of the lactobacilli strains tested, including clinic isolates from mothers of caries active kids, possessed strong capacity to colonize and form biofilms *in vitro* consistent with our early studies with *L. casei* (Van Houte et al., 1972; Filoche et al., 2004; Wen et al., 2010). When grown together with *S. mutans*, however, all lactobacilli showed major increases in biofilm formation, except *L. fermentum*, which reduced biofilms significantly. These results further suggest differences exist in intercellular interactions between *S. mutans* and different *Lactobacillus* spp. as well as their impact on the mixed-species consortium. In an early animal model study by Fitzgerald et al. (Fitzgerald et al., 1980), it was found that clinic isolates of *L. salivarius* and *L. fermentum* were able to induce significant carious activity in conventional hamsters but not on hamsters whose regular oral flora was depressed via polyantibiotics. This also indicates results of likely interactions between members of the indigenous oral flora and the lactobacilli infected.

Inter-species interactions are well documented in the oral cavity, which include adhesin-receptor mediated coadhesion and coaggregation, nutrient coupling, and metabolite mediated inhibitions (Kuramitsu et al., 2007). *S. mutans* possesses at least three Gtf enzymes that produce glucose polymers (better known as glucans) from sucrose, playing a central role in bacterial adherence and biofilm accumulation (Bowen and Koo, 2011). These Gtf proteins can also function as adhesins that bind directly to their adhesive products, the glucans. In addition, GtfB can also avidly bind to the surfaces of other bacterial species, including *Actinomyces vicosus, Candida albicans* and *L. casei* (Vacca-Smith and Bowen, 1998; Gregoire et al., 2011). Using radiolabeled GtfB, -C and -D in an *in vitro* binding assay, GtfB was found to possess the best capacity to bind to other bacteria tested (Vacca-Smith and Bowen, 1998). Consistently, the results presented here have also shown that GtfB can readily bind to *L. casei*, converting the cells to *de facto* glucan producers. *S. mutans* can directly bind to the *L. casei* cells (see also below), but as expected, significantly more *S. mutans* were found on the glucans synthesized by the GtfB that were bound on the surface of *L. casei* cells. These results further suggest that as an adhesin and an enzyme, GtfB is a major contributing factor to the enhanced biofilm formation by *L. casei* when grown with *S. mutans*. This is similar to what have recently been reported in *C. albicans* (Gregoire et al., 2011; Ellepola et al., 2017; Hwang et al., 2017; Kim et al., 2017), but to the best of our knowledge, has never been reported in *Lactobacillus* spp. before. In *C. albicans*, presence of *S. mutans'* GtfB was also recently shown to modulate expression of genes critical to cell morphology and biofilm formation (Ellepola et al., 2017). Studies are underway to elucidate the receptors GtfB binds to and if GtfB binding also influences other aspects of the *L. casei* physiology.

P1 protein is a high-affinity adhesin that *S. mutans* utilizes to colonize the tooth's salivary pellicle via interactions with the host scavenger receptor glycoprotein called GP340 or DMBT-1 (Jakubovics et al., 2005; Brady et al., 2011; Larson et al., 2011). As multi-functional adhesins, the AgI/II family of proteins, including SspA/B in *S. gordonii*, also interact with host proteins such as fibronectin and collagen and other bacteria, such as *Porphyromonas gingivalis* and *A. naeslundii* (Brady et al., 2011; Sullan et al., 2015). The results that P1 deficiency significantly diminishes the ability of *S. mutans* to facilitate *L. casei* biofilm formation also suggests that P1 is part of the underlying factors in *S. mutans-L. casei* interactions. While not surprising, this, too, has not yet been reported before. What receptors on *L. casei* cells P1 binds to and what regions of P1 are involved in binding await further investigation.

Like *S. mutans, Lactobacillus* spp. utilize a variety of sugars, although the end products of sugar fermentation vary between different species in response to environmental conditions, such as oxygen tension and sugar availability (Van Houte et al., 1972; Iwami and Yamada, 1985; Yamada et al., 1985; Iwami et al., 1992). Consistent with our previous studies of *L. casei* and *S. mutans* (Wen et al., 2010), co-cultivation of *S. mutans* with all *Lactobacillus* spp. tested, except *L. fermentum*, resulted in a lower culture pH than the respective mono-species cultures (data not shown). The reduced culture pH is likely a major underlying factor as to why *S. mutans* and probably, *L. casei* in dual-species biofilms had a significantly enhanced resistance to low pH than the respective mono-species cultures (Lemos et al., 2013). Interestingly, however, both *L. casei* and especially, *S. mutans* in dual-species displayed a drastic reduction in resistance to hydrogen peroxide, when compared to respective mono-species cultures. Co-current with the reduced stress tolerance was also the up-regulation of several genes known to play major roles in oxidative stress tolerance responses, as revealed by RNA-seq analysis. These include *sodA, nox1*, and *tpx*, which are all known to be inducible in response to oxidative stresses (Lemos et al., 2013). Among the up-regulated genes were also those for biosynthesis of mutanobactin, whose deficiency was shown to lead to major defects in oxidative stress tolerance response (Wu et al., 2010), and *clpB* and *clpX* for subunits of the Clp proteinase, members of the stress tolerance response pathways (Lemos et al., 2013). These results all suggest that *S. mutans* in dual-species with *L. casei* underwent some major oxidative stresses.

Both *S. mutans* and lactobacilli produce reactive oxygen species from oxygen metabolism, such as hydrogen peroxide, which can trigger the expression of an array of cytoprotective enzymes, including SodA and Nox1 (Ajdic et al., 2002; Bitoun et al., 2011; Lemos et al., 2013; Baker et al., 2014). However, unlike *S. mutans, L. casei* possesses two copies of genes for pyruvate oxidase, which in *S. gordonii* and *S. sanguinis* are responsible for hydrogen peroxide production (Kreth et al., 2005; Zheng et al., 2011a,b). Both SpxB expression and hydrogen peroxide production are modulated in response to cellular metabolic status via catabolite repression protein CcpA (Zheng et al., 2011a). As revealed by RNA-seq analysis, *S. mutans* seemingly alters sugar metabolism during growth with *L. casei* (see more details below), but it is unclear if similar phenomenon took place in *L. casei* in dual-species. It is likely that production of hydrogen peroxide by *L. casei* in the mixed-species consortium is up-regulated. If proven to be true, the elevated hydrogen peroxide will certainly be part of the underlying factors for the up-regulation of the stress genes and the increased susceptibility to hydrogen peroxide killing. Several oral lactobacilli are capable of producing bacteriocins effective against *S. mutans* causing cell envelope damage and death (Simark-Mattsson et al., 2007; Strahinic et al., 2007; Wu et al., 2015). However, none of the lactobacilli tested were shown to produce any visible antimicrobial activity against *S. mutans* under the conditions studied. On the other hand, *S. mutans* UA159 did produce some antimicrobials effective against *L. fermentum*, but not the other lactobacilli tested (Figure S4). Interestingly, of the genes identified as down-regulated in *S. mutans, comX* encodes the alternative Sigma factor, SigX that is well documented for its role in regulation of bacteriocins production (Khan et al., 2016). However, the actual impact of the altered SigX expression on *L. casei* and the mixed-species consortium as well as the underlying mechanism awaits further investigation.

Veillonellae are closely associated with *S. mutans* and other lactic acid bacteria in a community due to their reliance on lactic acids for carbon and energy sources. Consistent with Kara *et al*. on *V. parvula* (Kara et al., 2006, 2007; Luppens et al., 2008), the results presented here also showed that *V. dispar* alone did not form good biofilms, but co-cultivation of *S. mutans* and *V. dispar* yielded significantly more biofilms than the respective mono-species. Likely, such an enhancement can be in part attributed to the metabolic coupling between the two bacteria when grown together. In addition, as shown with *L. casei, S. mutans* GtfB and/or its adhesive products could also play a role.

Recent studies in several oral pathogens have generated evidence that growth in a community not only causes changes in community behavior, but may also elevate the pathogenic potential (Kara et al., 2006; Kuboniwa et al., 2009; Wen et al., 2010; Whitmore and Lamont, 2011; Falsetta et al., 2014; Sztajer et al., 2014; He et al., 2017). As revealed by RNA-seq analysis, co-cultivation with *L. casei* causes *S. mutans* to alter its transcriptional profile, including those involved in biofilm formation and oxidative stress responses. Of note, the down-regulation of *gtfB* and *spaP* (Table S2) by *S. mutans* in dual-species with *L. casei* is consistent with the results of our previous studies by RealTime-PCR (Wen et al., 2010). Interestingly, among the up-regulated genes are also those for Ftf and GtfD, and the down-regulated include a dextranase precursor. Unlike GtfB, GtfD produces primarily α[1,6]-linked water soluble glucose polymers, while Ftf synthesizes β(2,1 & 2,6)-linked fructose polymers, both of which are believed to primarily serve as storage of carbon and energy sources (Burne, 1998; Moye et al., 2014). These alterations suggest that *S. mutans* may undergo some major shifts in metabolic pathways when grown in consortium with *L. casei*. In support of this notion, several genes were identified (in <1.5-fold but still statistically significant) to encode proteins with roles in sugar metabolism, including *ccpA* (for carbon catabolite protein CcpA, a global regulator), *pfl* (for pyruvate formate lyase), *pflA* (pyruvate formate lyase activating enzyme), *msm* operon (SMU.876/80, for members of the multiple sugar metabolism system), and loci SMU.270/2 for ascorbate-specific PTS system EIIABC (Ajdic et al., 2002). The significant reduction of succinic acid and the increase of lactic acid in the culture medium of 24-h *S. mutans*-*L. casei* co-cultures, as compared to the respective mono-species cultures, also support the concept that interactions between these two major oral bacteria may lead to changes in sugar fermentation and acid production. Similar observations have also been made with *S. mutans* grown with *V. parvula* and more recently in *S. mutans* grown with *C. albicans* (Kara et al., 2006; He et al., 2017). However, how the sugar fermentation is altered and what effects of such alterations may have on *L. casei* and others in the consortium and ultimately, the pathogenicity of the community await further investigation.

In summary, these results have shown that *Lactobacillus* ssp. do not form good biofilms, although differences exist between different species. In mixed-species consortium, *S. mutans* can drastically enhance biofilm formation by all *Lactobacillus* spp. tested except *L. fermentum*, and both GtfB and P1 are major contributors to the enhanced biofilm formation by *L. casei*. On the other hand, *S. mutans* in dual-species with *L. casei* also displayed major reductions in oxidative stress tolerance and altered the expression of a large number of genes including those involved in biofilm formation and oxidative stress responses. These results suggest that interactions between *S. mutans* and *Lactobacillus* spp. vary between different species, although further investigation on what effects of such interactions may have on the plaque community and its cariogenicity is needed.

## Author contributions

SL, AD, and AJ performed most of the experiments and data analyses; JB and XX worked on metabolite analysis; SF and PSGC performed RNA-seq and post-sequencing analysis; PWC, YL, HK and ZW conceived the overall plan and interpreted the data; ZW wrote the draft; AJ help with revisions; YL and ZW wrote the final version.

### Conflict of interest statement

The authors declare that the research was conducted in the absence of any commercial or financial relationships that could be construed as a potential conflict of interest. The reviewer JK and handling Editor declared their shared affiliation.
