# Supplementary material for: Streptococcus mutans Displays Altered Stress Responses While Enhancing Biofilm Formation by Lactobacillus casei in Mixed-Species Consortium
Source: Front Cell Infect Microbiol. 2017 Dec 20;7:524. doi: 10.3389/fcimb.2017.00524 (PMC5742344; doi:10.3389/fcimb.2017.00524)
Supplement: Supplementary file 1 [file Presentation1.pdf]

Table S1. Modified Chemically Defined Medium (mFMC*)

Recipe for working medium:

1. Collect the major components (ml) (see table below)

Major component needed / Volume to be made	**1 liter**	**4 liters**	**6 liters**
1. Salt solution	100	400	600
2. 1 M Potassium Phosphate Buffer pH 7.6	25	100	150
3. Vitamin solution	20	80	120
4. Adenine Solution	5	20	30
5. Guanine Solution	5	20	30
6. Uracil Solution	10	40	60
7. Amino Acid Solution (in 0.1N HCl)	100	400	600
8. Tyrosine Solution (in 0.1N HCl)	100	400	600
9. Cysteine – HCl Solution	50	200	300
10. $NaHCO_3$ Solution	50	200	300
11. Ascobic acid solution	20	100	120
12. Polysorbate 80 (Tween 80)	1	4	6
13. Trace minerals	1	4	6
14. Additional nucleobases	5	20	30
15. Glucose and /or Sucrose	?	?	?
16. Double distilled H_2O	?	?	?

2. Adjust medium to pH 7.0 with NaOH
3. Filter sterilize using 0.2 µm filter units
4. Cover bottle with aluminum foil (some chemicals are light sensitive) and store in cold room.

mFMC stock solutions (All Stocks should be Filter-Sterilized):

1. **Salt solution**:

	**2L**	**4L**
$(NH_4)_2SO_4$	20g	40g
$MgSO_4$-$7H_2O$ (Magnesium sulfate)	4.0 (1.92g for anhydrous)	3.84 (anhydrous)
$FeSO_4$-$7H_2O$ (Ferrous sulfate)	0.8	1.6
$MnSO_4$-$2H_2O$ (Manganese sulfate)	0.03 (0.028 for monohydrous)	0.056 (monohydrous)
NaAc-$3H_2O$ (Sodium acetate trihydrate)	6.0	12g

Note: Filter solution using 2-3 sheets of Whatman paper to remove Fe ppt, before filter sterilization with 0.2µm filters.

2. **Potassium Phosphate Buffer solution:**

		(q.s to) 1**L**	**500ml**
base	a) 1M K_2HPO_4	174.18g (anhydrous)	87.09
acid	b) 1M KH_2PO_4	136.09g (anhydrous)	68.045

Make (a) and (b) up separately. Titrate the 1M K_2HPO_4 with 1M K_2HPO_4 to attain pH=7.6 (400ml w/ 50ml)

3. **Vitamin solution -- 1L**

	**In 1L ddH20**
Thiamine-HCl (B1)	0.2 g
Pyridoxamine-diHCl (B6)	0.05 g
Ca-Pantothenate (D-pantothenic acid)	0.2 g
Riboflavin (B2)	0.2 g
Nicotinamide (Niacinamide)	0.1 g
p-Aminobenzoic acid (PABA)	0.01 g
Biotin	0.005 g
Folic acid	0.005 g
Pyridoxine	0.05 g
Cyanocobalmin (B12)	0.05 g

Note: 1. Add concentrated NaOH drop-wise until vitamins are in solution.
2. Store solution in 100 ml aliquots in freezer (-20°C)

4. **Adenine solution -- 1L**
4 g adenine/liter q.s with dH_2O (Use conc. 10M NaOH to effect this solution)

5. **Guanine solution -- 1L**
4 g guanine/liter q.s with dH_2O (Use conc. 10M NaOH to effect this solution)

6. **Uracil solution -- 1L**
2 g uracil/liter q.s. withdH_2O (Use conc. 10M NaOH to effect this solution)

7. Amino Acid solution (L-isomers only) -- 2L

	**<u>2L 0.1N HCl</u>** (16.95mL glacial HCl in 2L dH_20)
glutamine	0.10 g
glutamic acid (room temp)	6.0 g
lysine (freezer 4 door)	2.2 g
aspartic acid	2.0 g
isoleucine	2.0 g
leucine	2.0 g
methionine	2.0 g
phenylalanine	2.0 g
threonine	2.0 g
valine	2.0 g
alanine	4.0 g
arginine	4.0 g
histidine	4.0 g
glycine	4.0 g
hydroxyproline	4.0 g
proline	4.0 g
tryptophan	4.0 g
Asparagine	4.0 g

Note: All amino acids are L form. Gently heat amino acids into solution. Can allocate and store at -20C for months.

8. Tyrosine Solution – 2L

4g/2L of 0.1N HCl (warm into solution)

9. Cysteine - HCl solution: – 1L

527 mg cysteine - HCl /liter dH_2O

10. $NaHCO_3$ solution: – 1L (q.s.)

10g $NaHCO_3$/100 mL dH_2O (0.5% conc. in final medium)

11. Ascobic acid:

2.5% sloution (or include in Vit mix), use 20 ml per liter

12. Polysorbate 80 (Tween 80):

0.2 ml per liter to working medium

13. Additional nucleobases:

2 g thymine, 2 g cytosine, 2 g cytidine, 2 g myo inositol

/liter q.s with dH_2O (Use conc. 10M NaOH to effect this solution), add 5 ml per liter

14. Trace minerals: 1 ml per liter of a trace element solution:

81.1 mM HCl, 0.8 mM $CoCl_2 \cdot 6H_2O$, 0.01 mM $CuCl_2 \cdot 2H_2O$, 0.1 mM H_3BO_3, 0.2 mM $Na_2MoO_4 \cdot 2H_2O$, 0.1 mM $NiCl_2$, 0.5 mM $ZnCl_2$

References:

1. **Elli M, Zink R, Rytz A, Reniero R, Morelli L.** 2000. Iron requirement of *Lactobacillus* spp. in completely chemically defined growth media. Journal of applied microbiology **88:**695-703.
2. **Terleckyj B, Willett NP, Shockman GD.** 1975. Growth of several cariogenic strains of oral streptococci in a chemically defined medium. Infect. Immun. **11:**649-655.
3. **Savijoki K, Suokko A, Palva A, Varmanen P.** 2006. New convenient defined media for [(35)S]methionine labelling and proteomic analyses of probiotic lactobacilli. Letters in applied microbiology **42:**202-209.
4. **Wegkamp A, de Vos WM, Smid EJ.** 2009. Folate overproduction in *Lactobacillus plantarum* WCFS1 causes methotrexate resistance. FEMS microbiology letters **297:**261-265.

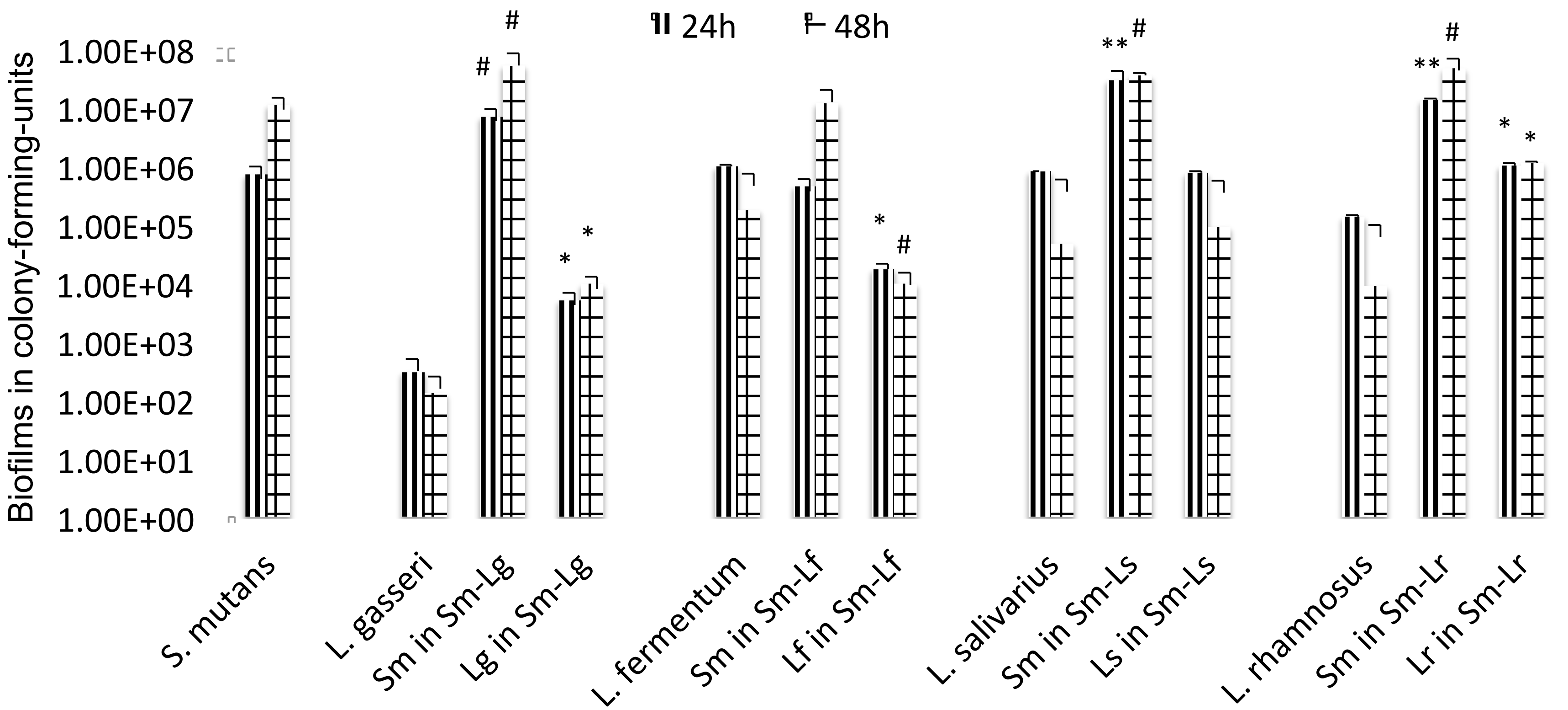

Fig. S1. 24 and 48-hour biofilms of *L. gasseri* (Lg), *L. fermentum* (Lf), *L. salivarius* (Ls) and *L. rhamnosus* (Lr) in mono-species and dual-species with *S. mutans* (Sm) grown in BM plus glucose (18 mM) and sucrose (2 mM) on glass slides vertically deposited in 50 ml Falcon tubes. Data represent the average (±standard deviation in error bars) of at least three separate experiments, with #, * and ** indicating statistical difference at *P*<0.05, 0.01 and 0.001, respectively, when compared to the respective mono-species biofilms.

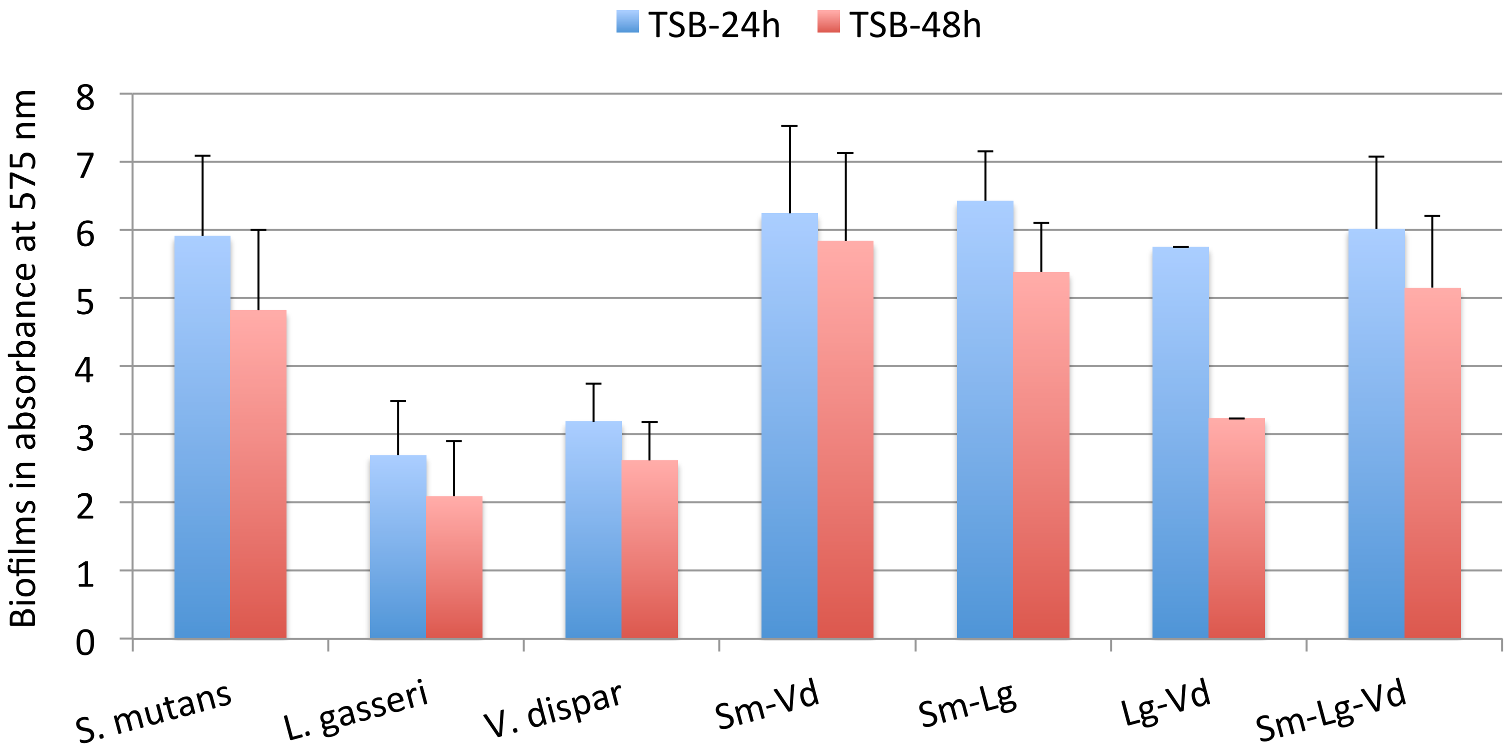

Fig. S2a. 24 and 48-hour biofilms of *S. mutans* (Sm), *L. gasseri* (Lg), and *V. dispar* (Vd) when grown in tryptic soy broth (TSB) either alone or in mixed-species for 24 and 48 hours in 96 well plates. Total biofilms were stained using 0.1% crystal violet and measured by a spectrophotometry at 575 nm. Data represent the average (±standard deviation in error bars) of three separate experiments.

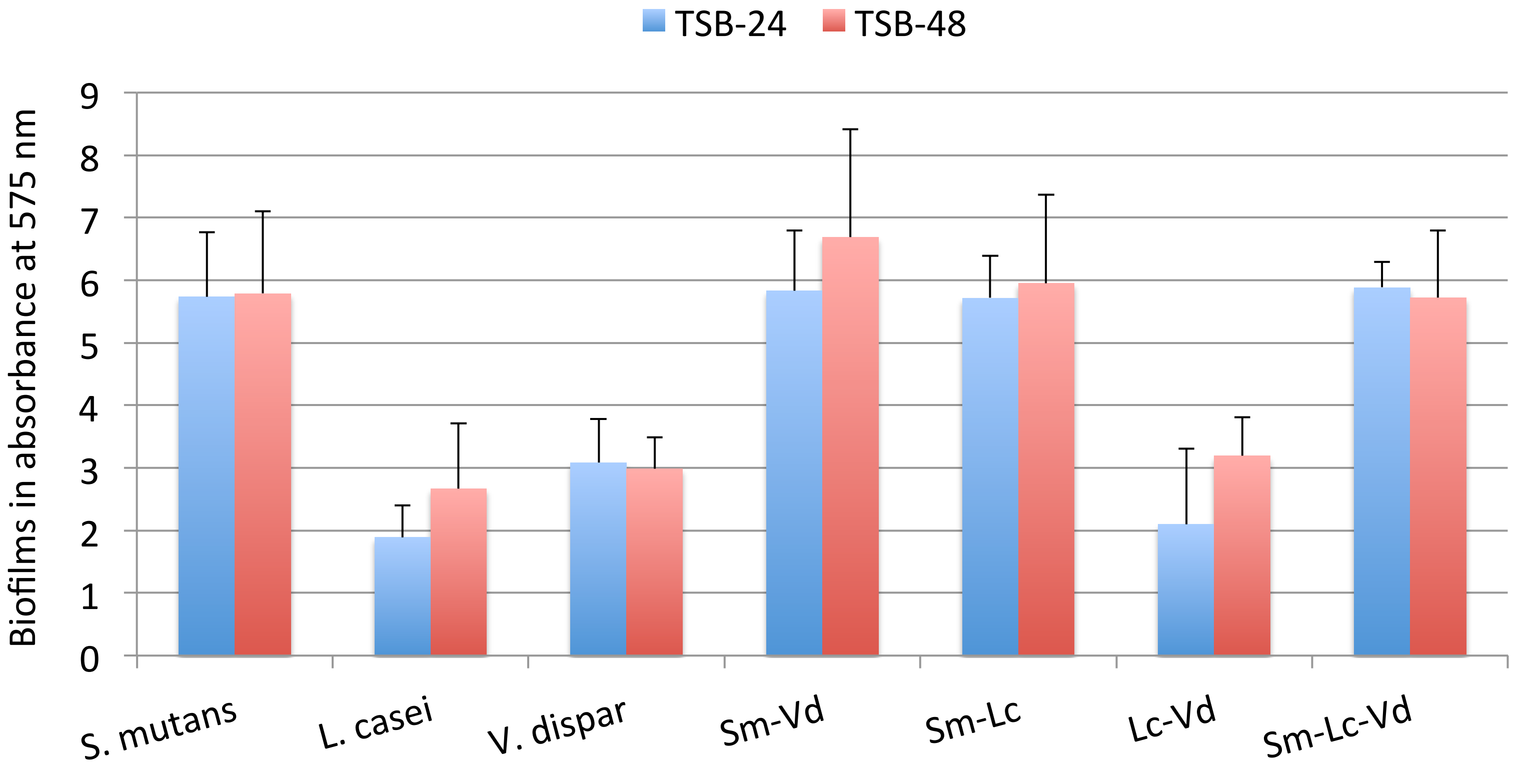

Fig. S2b. 24 and 48-hour biofilms of *S. mutans* (Sm), *L. casei* (Lc), and *V. dispar* (Vd) when grown in tryptic soy broth (TSB) either alone or in mixed-species for 24 and 48 hours in 96 well plates. Total biofilms were stained using 0.1% crystal violet and measured by a spectrophotometry at 575 nm. Data represent the average (±standard deviation in error bars) of three separate experiments.

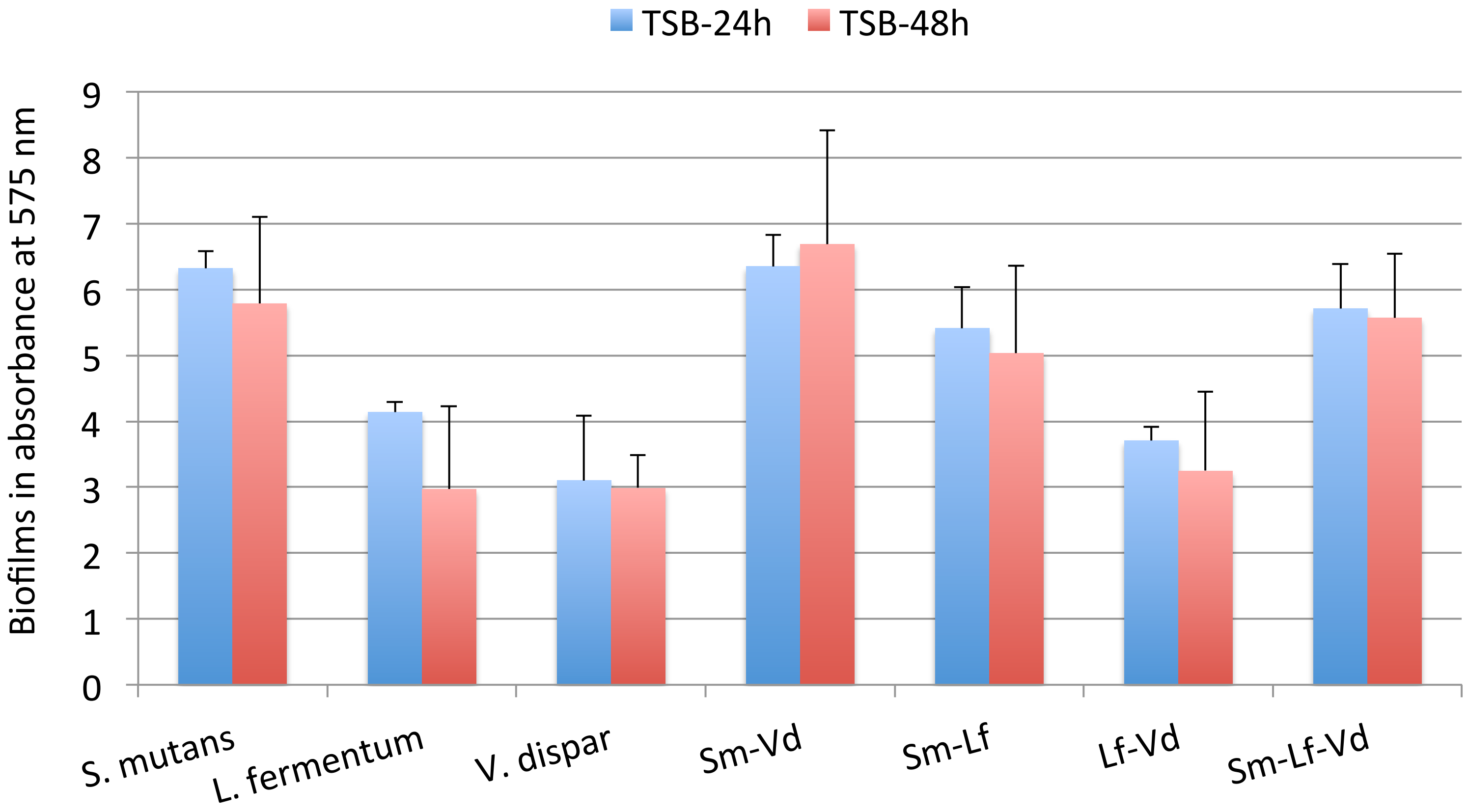

Fig. S2c. 24 and 48-hour biofilms of *S. mutans* (Sm), *L. fermentum* (Lf), and *V. dispar* (Vd) when grown in tryptic soy broth (TSB) either alone or in mixed-species for 24 and 48 hours in 96 well plates. Total biofilms were stained using 0.1% crystal violet and measured by a spectrophotometry at 575 nm. Data represent the average (±standard deviation in error bars) of three separate experiments.

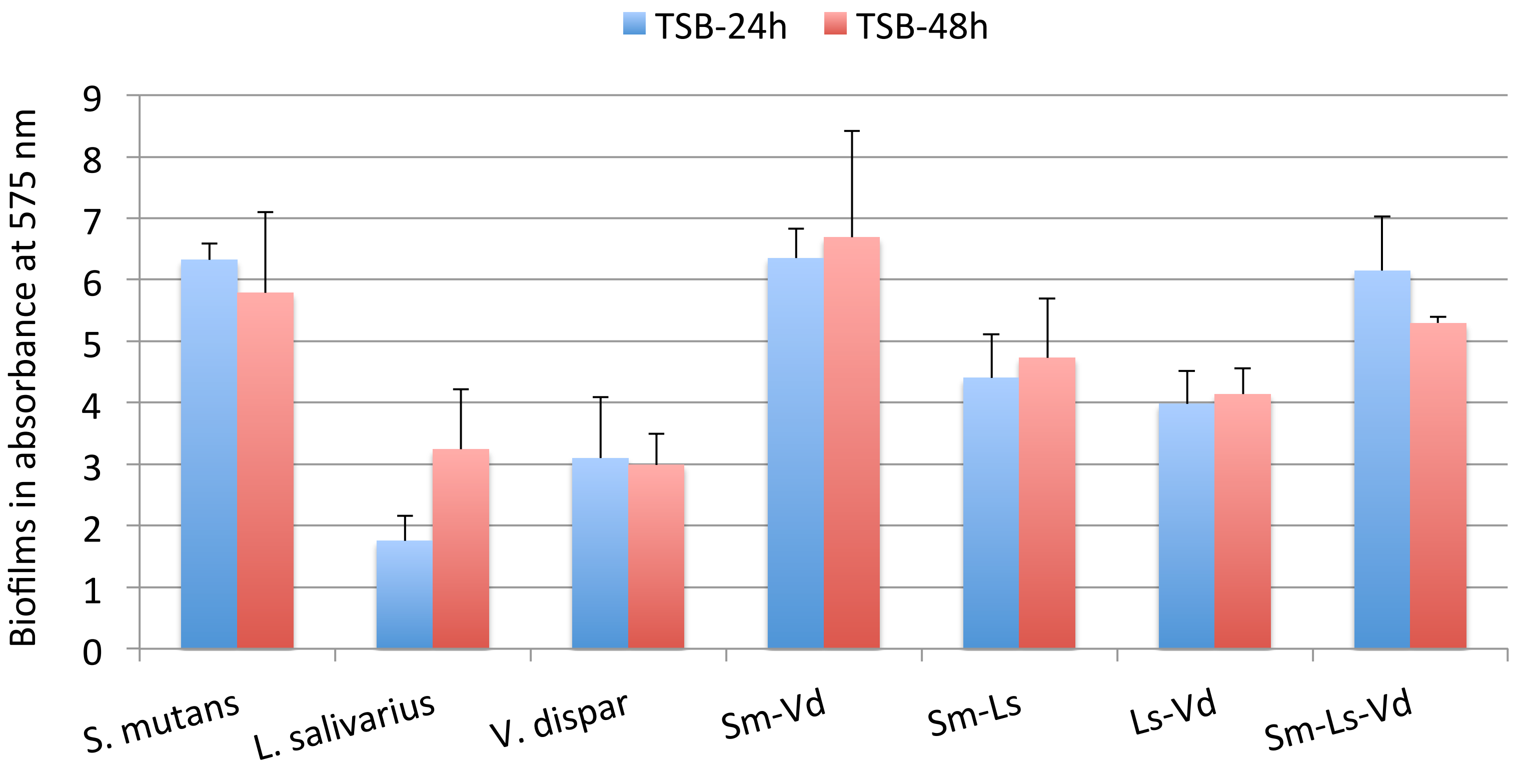

Fig. S2d. 24 and 48-hour biofilms of *S. mutans* (Sm), *L. salivarius* (Ls), and *V. dispar* (Vd) when grown in tryptic soy broth (TSB) either alone or in mixed-species for 24 and 48 hours in 96 well plates. Total biofilms were stained using 0.1% crystal violet and measured by a spectrophotometry at 575 nm. Data represent the average (±standard deviation in error bars) of three separate experiments.

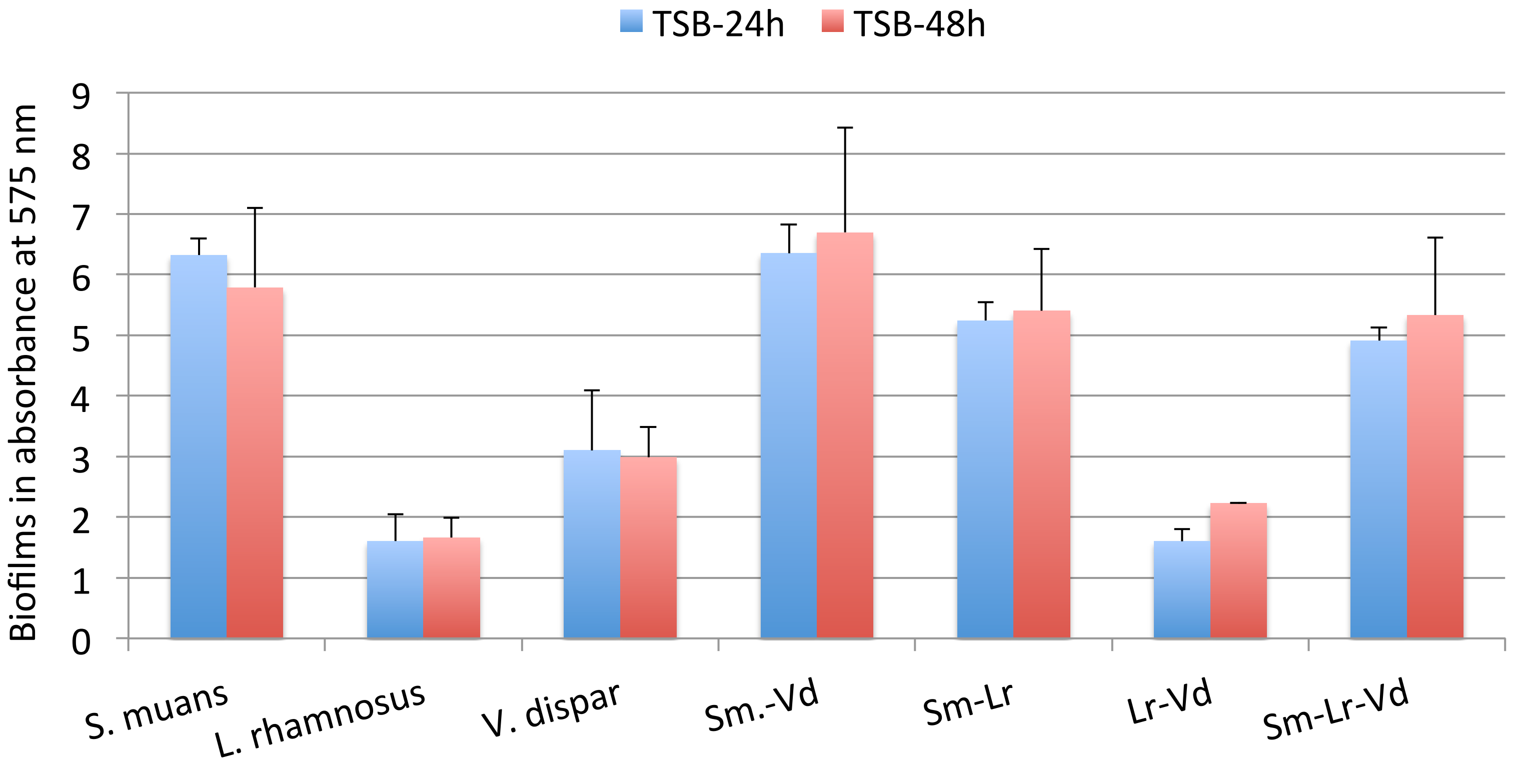

Fig. S2e. 24 and 48-hour biofilms of *S. mutans* (Sm), *L. rhamnosus* (Lr), and *V. dispar* (Vd) when grown in tryptic soy broth (TSB) either alone or in mixed-species for 24 and 48 hours in 96 well plates. Total biofilms were stained using 0.1% crystal violet and measured by a spectrophotometry at 575 nm. Data represent the average (±standard deviation in error bars) of three separate experiments.

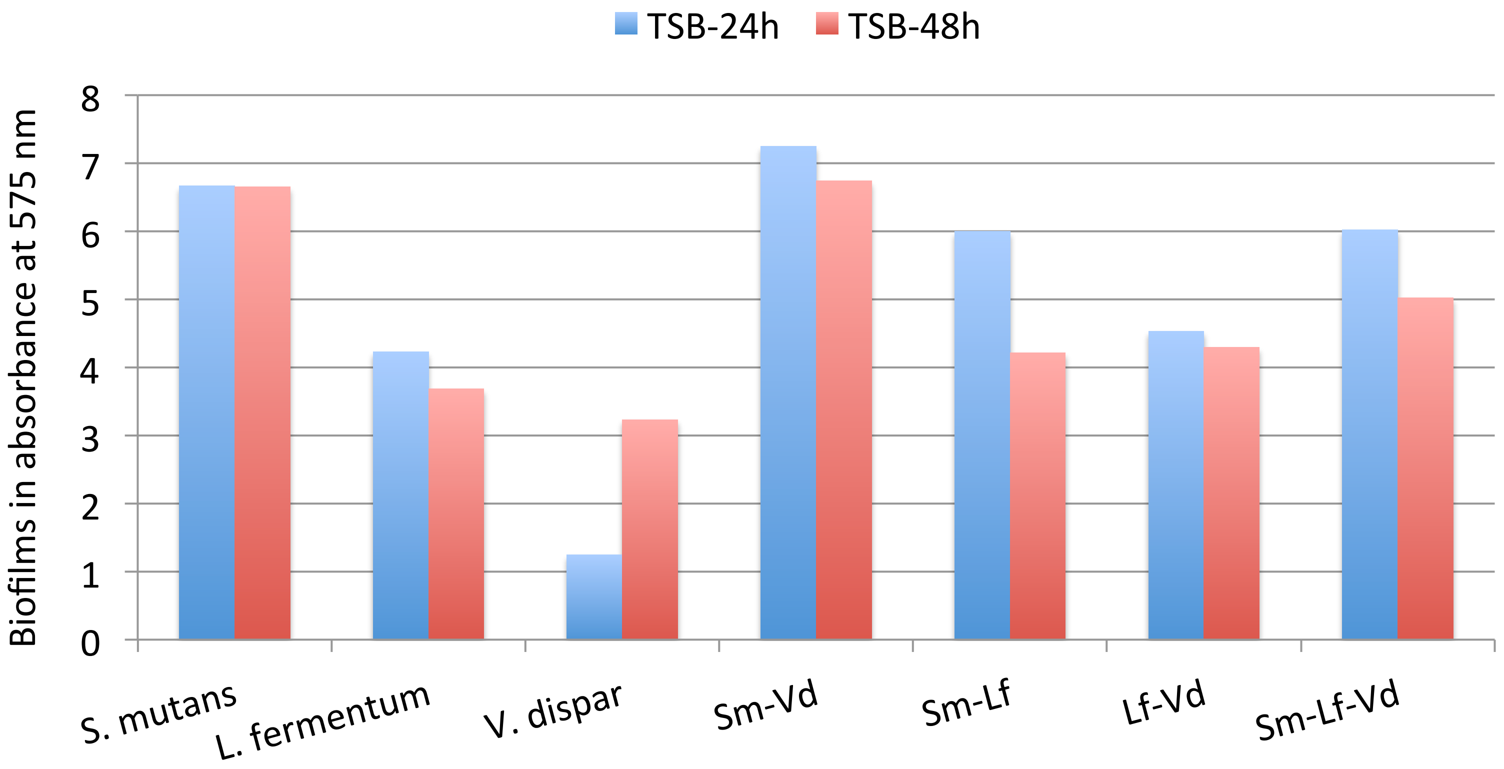

Fig. S2f. 24 and 48-hour biofilms of *S. mutans* (Sm), *L. fermentum* clinic isolate M87-28 (Lf), and *V. dispar* (Vd) when grown in tryptic soy broth (TSB) either alone or in mixed-species for 24 and 48 hours in 96 well plates. Total biofilms were stained using 0.1% crystal violet and measured by a spectrophotometry at 575 nm. Data represent the average of three replicate from one experiment.

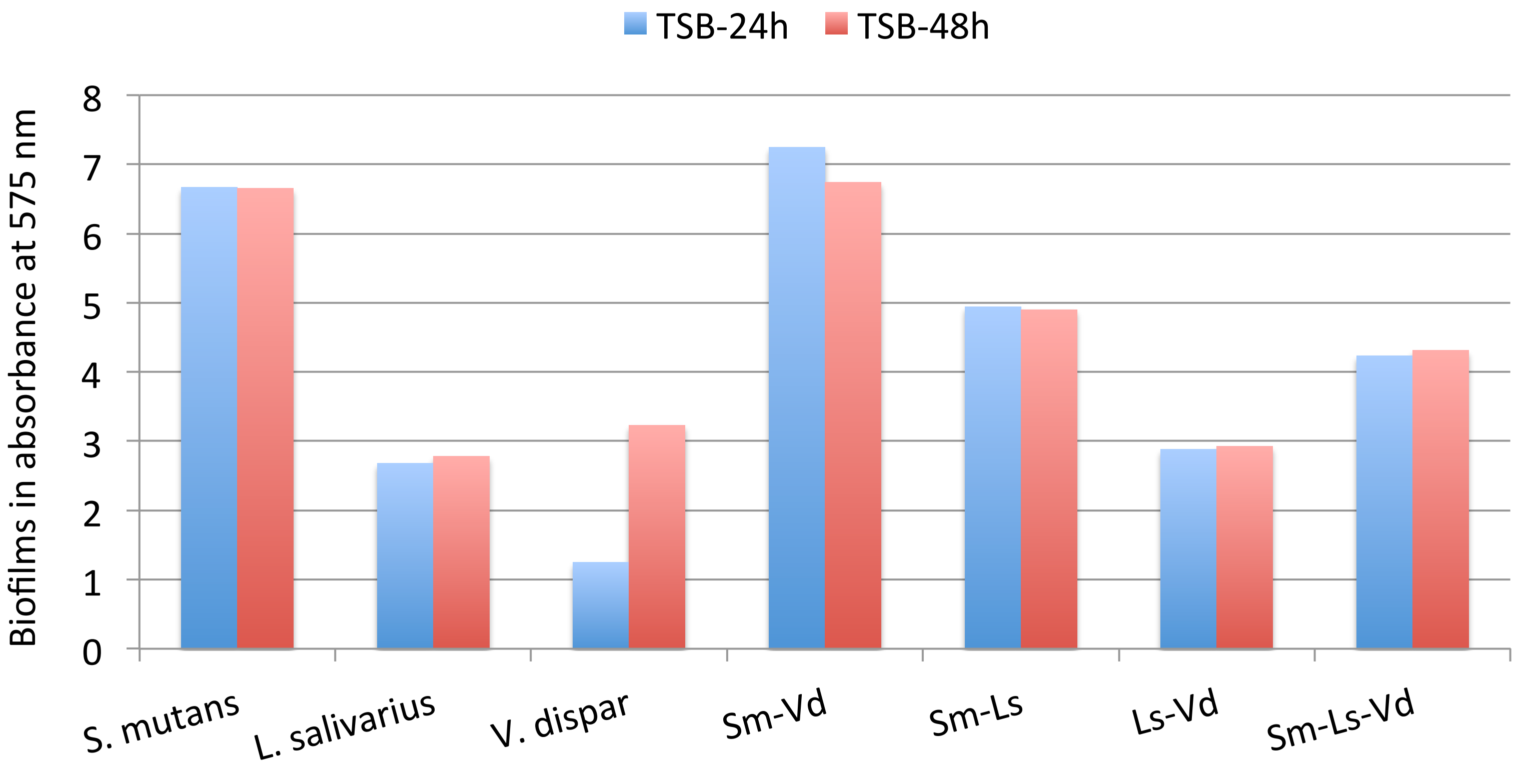

Fig. S2g. 24 and 48-hour biofilms of *S. mutans* (Sm), *L. salivarius* clinic isolate M87-8 (Ls), and *V. dispar* (Vd) when grown in tryptic soy broth (TSB) either alone or in mixed-species for 24 and 48 hours in 96 well plates. Total biofilms were stained using 0.1% crystal violet and measured by a spectrophotometry at 575 nm. Data represent the average of three replicates of one experiment.

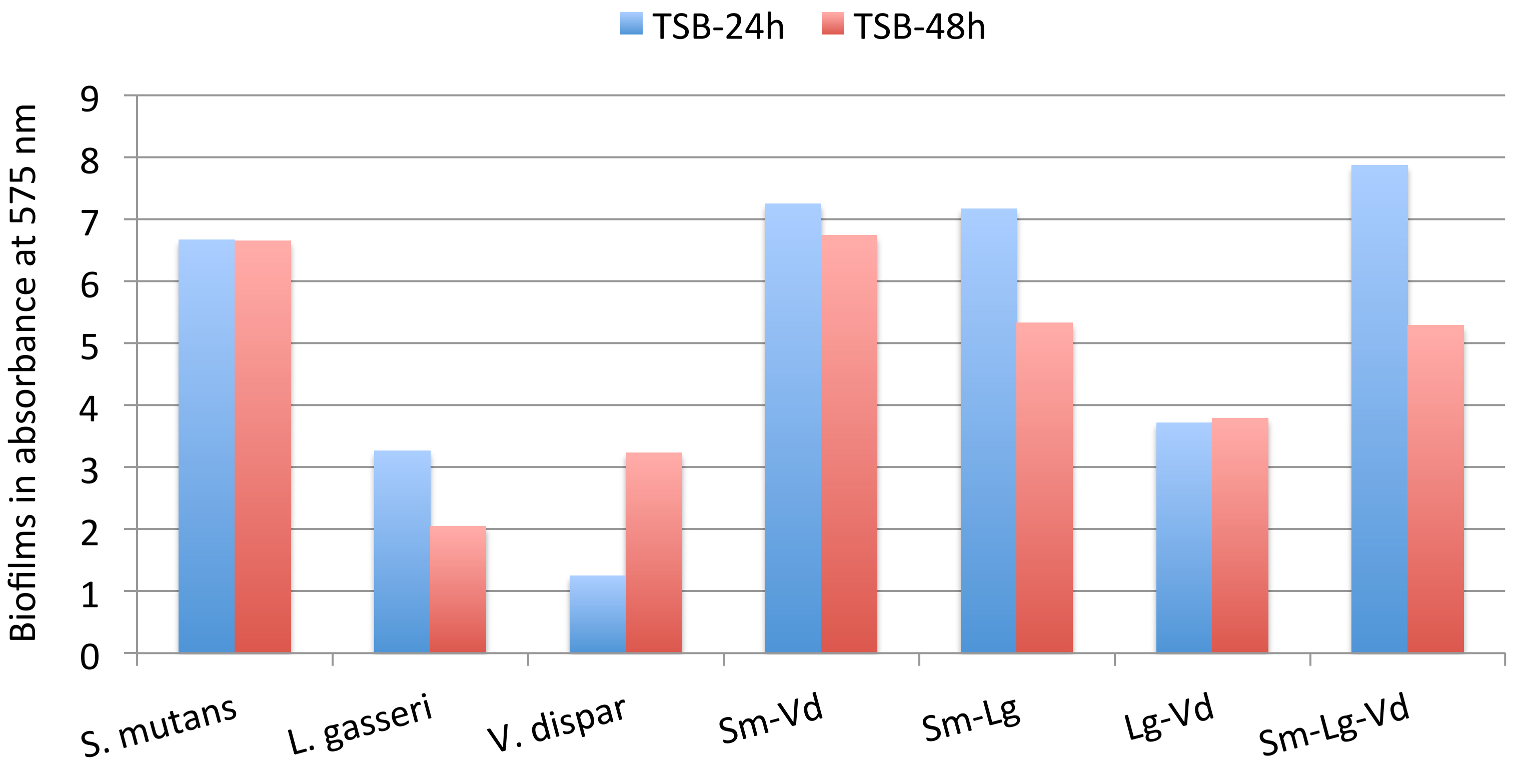

Fig. S2h. 24 and 48-hour biofilms of *S. mutans* (Sm), *L. gasseri* clinic isolate M57-23(Lg), and *V. dispar* (Vd) when grown in tryptic soy broth (TSB) either alone or in mixed-species for 24 and 48 hours in 96 well plates. Total biofilms were stained using 0.1% crystal violet and measured by a spectrophotometry at 575 nm. Data represent the average of three replicates of one experiment.

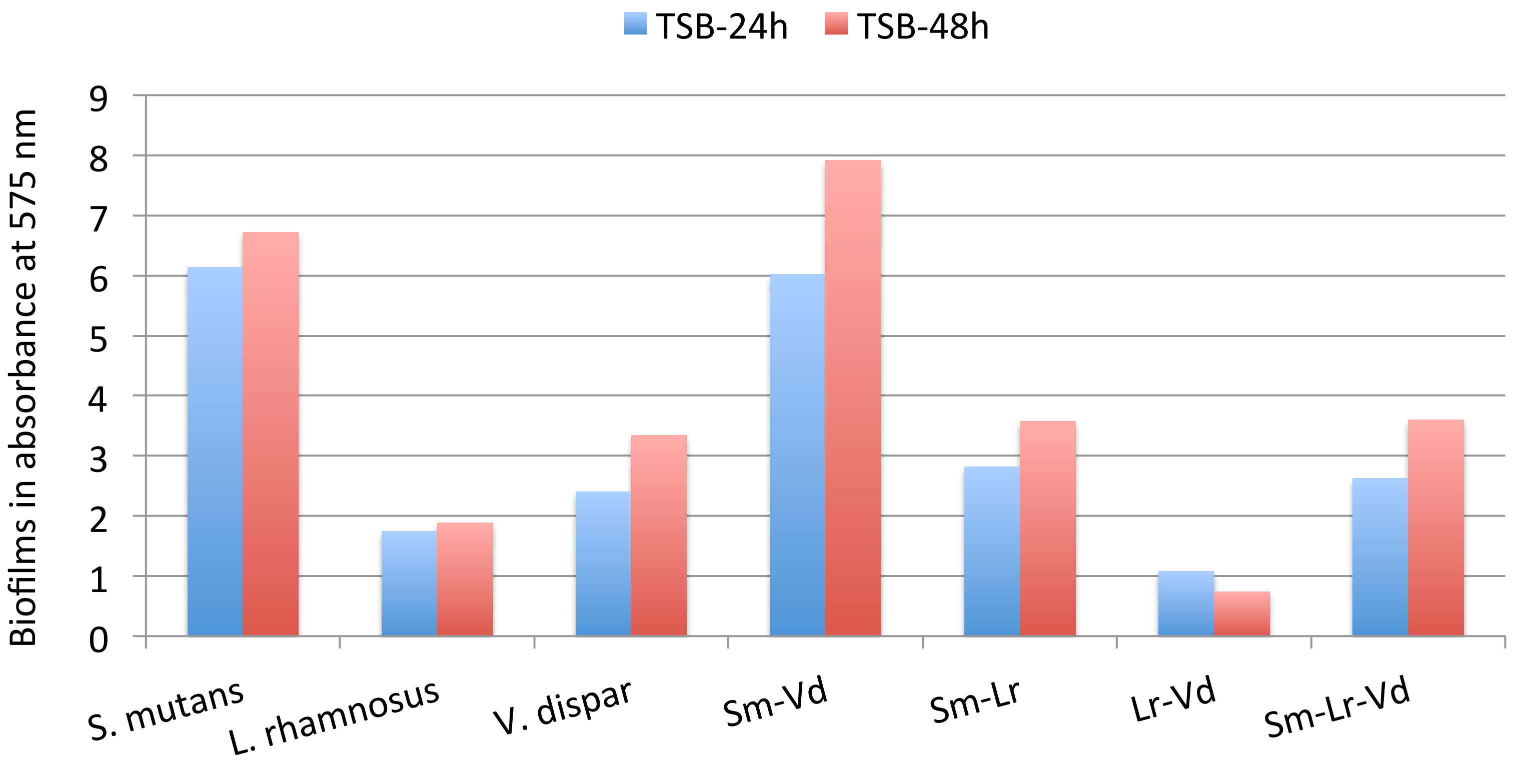

Fig. S2i. 24 and 48-hour biofilms of *S. mutans* (Sm), *L. rhamnosus* clinic isolate M87-26 (Lr), and *V. dispar* (Vd) when grown in tryptic soy broth (TSB) either alone or in mixed-species for 24 and 48 hours in 96 well plates. Total biofilms were stained using 0.1% crystal violet and measured by a spectrophotometry at 575 nm. Data represent the average of three replicates from one experiment.

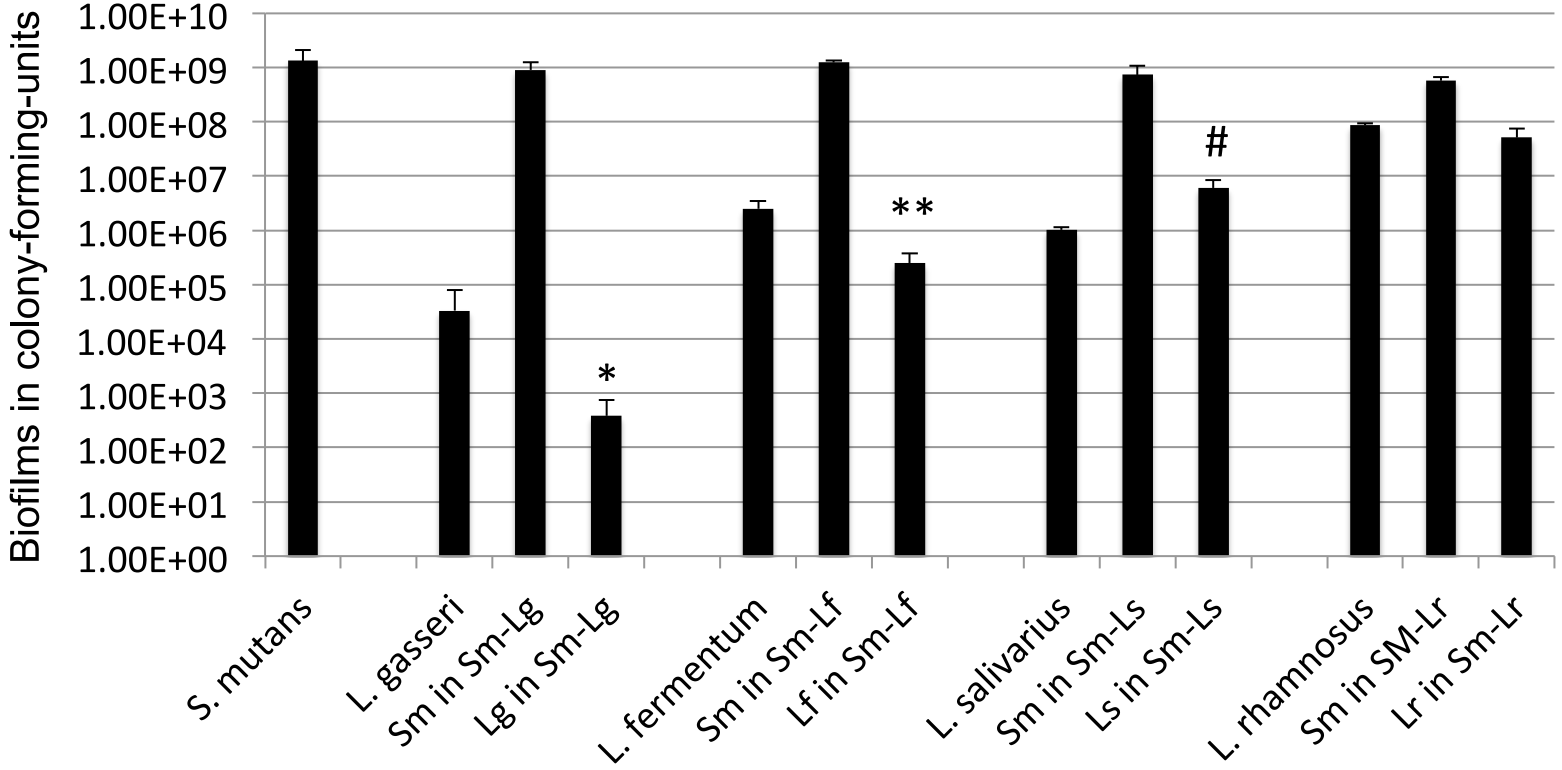

Fig. S3. 5 day biofilms in TSB. Biofilms of *L. gasseri* (Lg), *L. fermentum* (Lf), *L. salivarius* (Ls) and *L. rhamnosus* (Lr) in mono-species and dual-species with *S. mutans* (Sm) grown in TSB broth on glass slides vertically deposited in 50 ml Falcon tubes. Data represent the average (±standard deviation in error bars) of at least three separate experiments, with *, **, and # indicating statistical difference at $P<0.001$, 0.01 and 0.05, respectively, when compared to the respective mono-species biofilms.

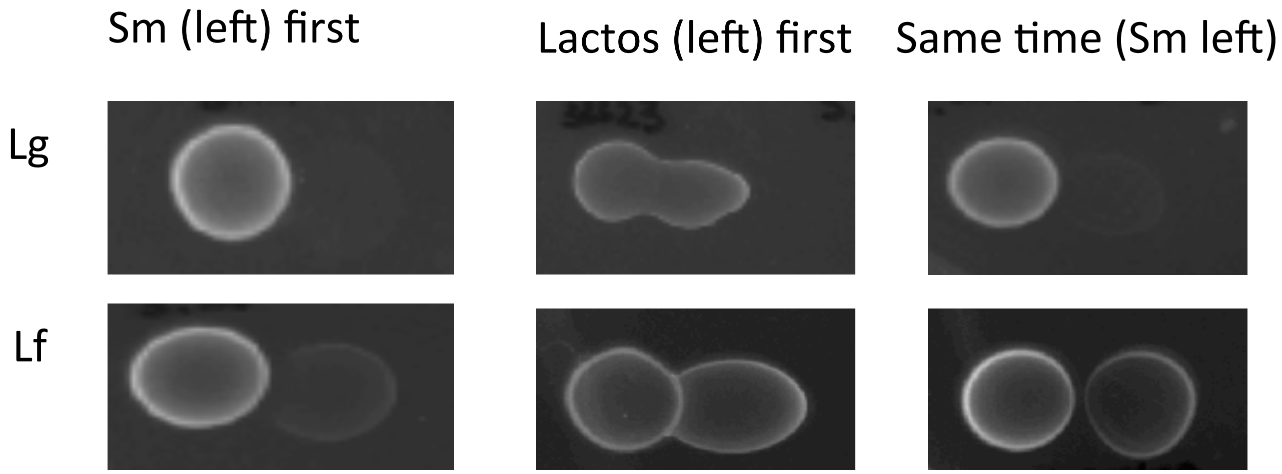

Fig. S4. Competition assays between *S. mutans* (Sm) and *L. gasseri* (Lg) and *L. fermentum* (Lf). Competition assays on half-strength BHI plates were done with either *S. mutans* spot first, lactobacillus (lactos) first, or at the same time. Results show that *S. mutans* produces inhibitory activity against *L. gasseri* when *S. mutans* was spotted first or at the same time, but not when *L. gasseri* was spot first. *S. mutans* was also shown to produce antimicrobial activity against *L. fermentum* when *S. mutans* was spotted first. No apparent antimicrobial activities were observed with other bacteria tested.

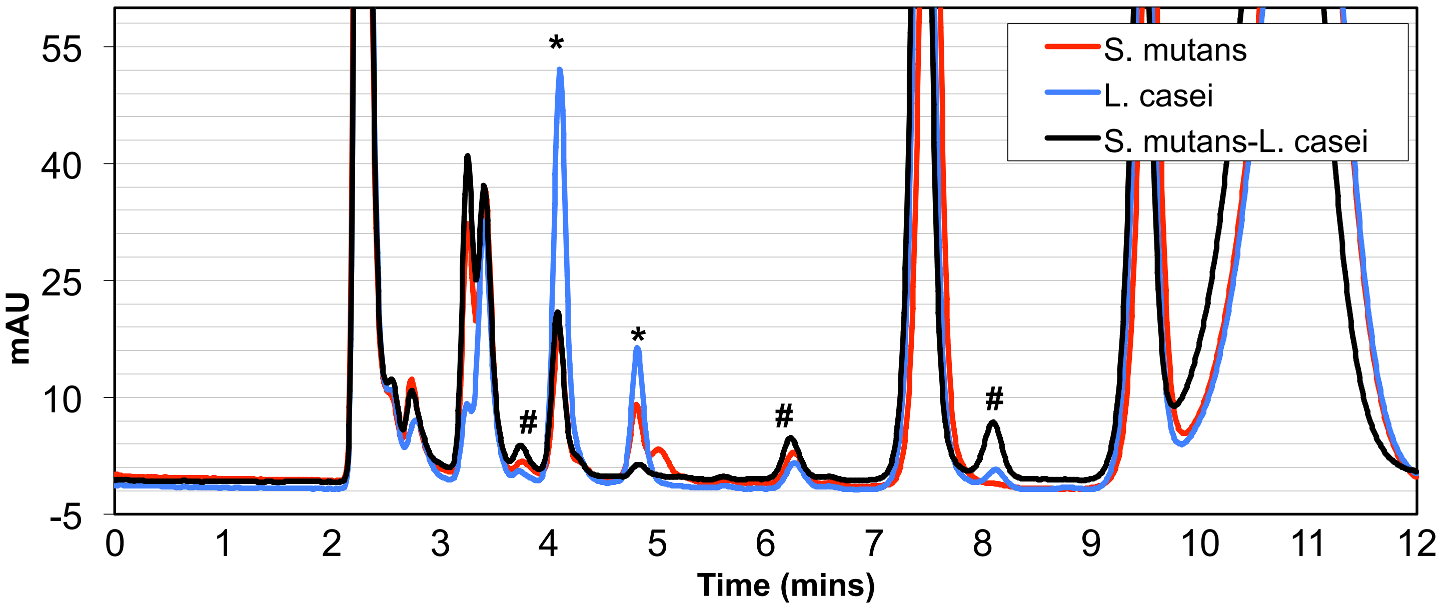

Fig. S5. UV-HPLC analysis of organic acids from mono- and dual species biofilms of *S. mutans* and *L. casei* grown in mFMC with 18 mM glucose and 2 mM sucrose for 24- and 48-hours in a 5% CO_2 chamber at 37°C. The sample supernates were filtered and diluted 10-fold in 40 mM Na_2SO_4, pH 2.65, and passed over a Acclaim™ OA 3 µm column at 0.21 mL/min of 40 mM Na_2SO_4 mobile phase at 30°C on a Agilent 1100 Series UV/HPLC. Samples were detected at 210 nm. Major metabolic changes (#, up; *, down) were apparent when they were grown together as compared to the respective mono-species cultures. Of the acids analyzed, succinic acid (RT=4.75 min) was shown to be down by 4-fold. Other more subtle changes were also identified for lactic acid (RT=3.21 min), α-ketoglutaric acid (RT=3.18 min), fumaric acid (RT=3.4 min), and citric acid (RT=4.03 min). Data is representative of two independent experiments.

Table S2. Genes identified by RNA-seq

Gene	Description	Ratio (D/M)	EdgeR_PValue	Deseq_pvalue
SMU_1346	putative_thioesterase_BacT	2.148699312	2.98E-12	3.21E-14
SMU_1117	NADH_oxidase_%28H2O-forming%29 (Nox1)	2.093387363	5.76E-26	5.37E-25
SMU_1342	putative_bacitracin_synthetase_1%3B_BacA	1.919121505	5.73E-15	1.68E-14
SMU_1340	putative_surfactin_synthetase	1.717681521	2.62E-10	5.08E-11
SMU_2028	beta-D-fructosyltransferase	1.717215725	7.67E-26	1.57E-66
SMU_924	lipid_hydroperoxide_peroxidase	1.670290341	1.54E-12	1.47E-12
SMU_270	unknown	1.666499631	0.000835434	0.033024915
SMU_1339	putative_bacitracin_synthetase	1.649627841	6.93E-08	1.07E-10
SMU_629	unknown (Sod, sodA, superoxide dismutase, Mn-dependent)	1.599778511	2.48E-08	7.27E-08
SMU_910	glucosyltransferase-S	1.499260046	1.98E-14	7.90E-29
SMU_463	putative_thioredoxin_reductase_%28NADPH%29	1.451377735	1.19E-07	1.71E-07
SMU_1814	putative_histidine_kinase%2C_ScnK-like_protein	1.449282767	3.75E-11	5.65E-17
SMU_271	putative_PTS_system_transporter_subunit_IIB	1.424139943	4.92E-09	4.43E-11
SMU_272	putative_PTS_system_transporter_subunit_IIA	1.405562718	2.44E-05	3.58E-10
SMU_838	unknown	1.400886043	4.54E-07	1.16E-06
SMU_1091	putative_cell_wall_protein%2C_WapE	1.354719234	6.09E-08	7.40E-11
SMU_1815	putative_response_regulator%3B_ScnR-like_protein	1.352200578	0.001398819	1.30E-05
SMU_402	pyruvate_formate-lyase	1.327348305	1.53E-05	6.30E-06
SMU_1561	putative_potassium_uptake_system_protein_TrkB	1.318066038	0.000180268	0.000275494
SMU_1562	putative_potassium_uptake_protein_TrkA	1.315586363	1.65E-06	1.76E-07
SMU_1928	putative_ABC_transporter_permease	1.275671425	0.007531019	6.74E-07
SMU_876	putative_MSM_operon_regulatory_protein	1.272128984	0.019124636	0.000363458
SMU_772	glucan-binding_protein_D	1.267441598	4.61E-05	2.86E-09
SMU_1650	putative_endonuclease_III_%28DNA_repair%29	1.261506581	0.020611862	0.000409438
SMU_1563	putative_cation-transporting_P-type_ATPase_PacL	1.259554032	0.002857586	0.000948009
SMU_1869	putative_thioredoxin	1.259412307	0.000744883	0.000160245
SMU_1916	putative_histidine_kinase_of_the_competence_regulon%2C_ComD	1.258827914	0.003327658	5.82E-06
SMU_1590	cytoplasmic_alpha-amylase	1.247300102	0.003004547	0.000999758
SMU_1322	acetoin_reductase	1.244515728	2.68E-05	9.85E-14

SMU_994	ribonuclease_HII	1.235628472	4.88E-05	1.54E-08
SMU_885	galactose_operon_repressor_GalR	1.232955852	0.006227263	1.69E-05
SMU_1077	putative_phosphoglucomutase	1.22849538	0.000871175	5.31E-05
SMU_1603	putative_lactoylglutathione_lyase	1.210569015	0.000437044	2.38E-06
SMU_1115	L-lactate_dehydrogenase	1.203328675	0.000623951	4.70E-09
SMU_495	glycerol_dehydrogenase	1.202196961	0.001202255	8.95E-07
SMU_1601	6-phospho-beta-glucosidase	1.202101779	0.001835259	1.02E-05
SMU_1032	putative_transposon_integrase%3B_Tn916_ORF3-like	1.199231947	0.040130593	8.77E-06
SMU_1170	putative_cytochrome_C_biogenesis_protein	1.177203914	0.011000339	0.000315079
SMU_1095	putative_choline_ABC_transporter%2C_osmoprotectant_binding_protein	1.171966104	0.002156088	9.91E-07
SMU_1515	hypothetical_protein	1.169213767	0.006071256	0.000352212
SMU_2117	putative_osmoprotectant_ABC_transporter_permease	1.168518202	0.003946453	6.45E-05
SMU_2118	putative_ABC_transporter_glycine_betaine%2Fcarnitine%2Fcholine-binding_protein	1.158869769	0.004675685	4.48E-06
SMU_135	putative_transcriptional_regulator	1.158539577	0.011276745	5.33E-05
SMU_1516	putative_histidine_kinase_CovS	1.155610746	0.008343137	0.000186773
SMU_562	ATP-dependent_protease_ClpE	1.149381127	0.014628861	0.00040772
SMU_987	cell_wall-associated_protein_WapA	1.149271871	0.009114396	0.000155585
SMU_1075	phosphopantothenoylcysteine_decarboxylase	1.140882295	0.019067146	4.30E-05
SMU_1839	mannose-6-phosphate_isomerase	1.136838858	0.017183315	0.000134284
SMU_1126	unknown	1.135549074	0.03468349	1.34E-05
SMU_888	UDP-galactose_4-epimerase_GalE	1.131194013	0.023499921	5.29E-05
SMU_1188	putative_signal_peptidase	1.129971081	0.013403014	2.48E-06
SMU_1050	unknown	1.129613172	0.013747805	1.80E-05
SMU_1020	putative_citrate_lyase_CilB%2C_citryl-CoA_lyase_subunit_beta	1.123726036	0.116705634	0.000136045
SMU_1564	putative_glycogen_phosphorylase	1.100207533	0.05783452	0.00037338
SMU_1023	unknown	1.097796412	0.081387299	0.000981846
SMU_1819	aspartyl%2Fglutamyl-tRNA_amidotransferase_subunit_B	1.092315406	0.078678616	0.000732626
SMU_2036	putative_peptidase	1.077247792	0.133071007	0.000446798
SMU_1581	DNA_polymerase_III_subunits_gamma_and_tau	-1.190437396	0.000681307	0.003306267
SMU_1860	30S_ribosomal_protein_S6	-1.195903338	0.00038034	0.005409829
SMU_610	cell_surface_antigen_SpaP	-1.198710492	0.000995092	0.004188609

SMU_231	unknown	-1.203832724	0.000744254	0.001389068
SMU_1858	30S_ribosomal_protein_S18	-1.204515019	0.000301529	0.001979924
SMU_1783	prolyl-tRNA_synthetase	-1.2047586	0.000119942	0.000311648
SMU_1859	single-stranded_DNA-binding_protein	-1.20503223	0.000394331	0.000230033
SMU_1770	valyl-tRNA_synthetase	-1.213898552	0.000571101	0.00217262
SMU_949	ATP-dependent_protease_ATP-binding_subunit_ClpX	-1.215754744	0.000141076	7.42E-06
SMU_1736	acetyl-CoA_carboxylase_biotin_carboxylase_subunit	-1.218733867	0.000139337	5.26E-07
SMU_2018	30S_ribosomal_protein_S17	-1.219176689	0.00057638	0.001750256
SMU_716	putative_peptidoglycan_branched_peptide_synthesis_protein_MurN	-1.221635353	0.000560685	0.002478737
SMU_1572	UDP-N-acetylglucosamine_1-carboxyvinyltransferase	-1.223874924	3.68E-05	0.000198542
SMU_1785	putative_phosphatidate_cytidylyltransferase_synthase	-1.225856377	0.000523875	0.001029555
SMU_671	citrate_synthase	-1.225947394	7.44E-05	0.000997185
SMU_2101	aspartyl-tRNA_synthetase	-1.226866418	0.000138583	0.000607898
SMU_2022	50S_ribosomal_protein_L22	-1.22739472	0.000604413	0.000427639
SMU_2015	50S_ribosomal_protein_L5	-1.232783932	0.000144074	8.21E-06
SMU_1919	hypothetical_protein	-1.234133728	0.005694052	0.000241441
SMU_2021	30S_ribosomal_protein_S3	-1.234899499	0.000175139	0.001134906
SMU_672	unknown	-1.238014562	1.04E-05	2.46E-06
SMU_1955	co-chaperonin_GroES	-1.239499703	3.57E-05	1.79E-05
SMU_1123	deoxyribose-phosphate_aldolase	-1.240940736	0.000342588	0.006980746
SMU_2016	50S_ribosomal_protein_L24	-1.24673606	4.37E-05	3.92E-06
SMU_2012	30S_ribosomal_protein_S8	-1.248954399	7.48E-06	1.58E-05
SMU_1396	glucan-binding_protein_GbpC	-1.250048875	4.48E-05	0.000103439
SMU_1737	%283R%29-hydroxymyristoyl-ACP_dehydratase	-1.250994979	8.00E-06	2.47E-07
SMU_1741	putative_malonyl-CoA_%28acyl-carrier-protein%29_transacylase	-1.257075078	5.04E-06	2.92E-06
SMU_779	3-dehydroquinate_synthase	-1.257306131	6.00E-05	9.79E-05
SMU_233	ketol-acid_reductoisomerase	-1.259770815	6.89E-06	2.39E-05
SMU_1122	cytidine_deaminase	-1.262414211	0.000234504	0.000160971
SMU_396	putative_glycerol_uptake_facilitator_protein	-1.263943616	0.000688212	0.012760079
SMU_2014	30S_ribosomal_protein_S14	-1.265006048	0.008034041	0.000432331
SMU_2009	30S_ribosomal_protein_S5	-1.265370567	2.16E-06	6.03E-09

SMU_456	phospho-N-acetylmuramoyl-pentapeptide-transferase	-1.26655216	2.85E-05	1.31E-06
SMU_1739	3-oxoacyl-%28acyl_carrier_protein%29_synthase_II	-1.268802398	1.20E-05	7.15E-07
SMU_1735	acetyl-CoA_carboxylase_subunit_beta	-1.270225727	1.88E-06	6.39E-09
SMU_2020	50S_ribosomal_protein_L16	-1.272285408	1.00E-05	0.000172171
SMU_2006	preprotein_translocase_subunit_SecY	-1.272344739	3.53E-06	3.06E-08
SMU_1204	DNA_topoisomerase_IV_subunit_A	-1.274079803	0.000197813	0.003880194
SMU_1834	alanine_racemase	-1.27423748	0.000461375	0.005236655
SMU_780	unknown	-1.275102039	6.87E-05	0.000400912
SMU_1738	putative_biotin_carboxyl_carrier_protein_of_acetyl-CoA_carboxylase	-1.275749483	7.26E-07	3.72E-07
SMU_970	unknown	-1.278832816	0.007417606	0.000975664
SMU_455	putative_penicillin-binding_protein_2X	-1.279725412	3.49E-07	4.80E-10
SMU_1740	3-ketoacyl-%28acyl-carrier-protein%29_reductase	-1.29089532	3.63E-07	1.11E-08
SMU_1744	3-oxoacyl-%28acyl_carrier_protein%29_synthase_III	-1.292098597	8.45E-06	0.000165245
SMU_2011	50S_ribosomal_protein_L6	-1.294456604	2.85E-07	6.48E-09
SMU_2025	50S_ribosomal_protein_L3	-1.298200268	1.81E-07	1.19E-06
SMU_2017	50S_ribosomal_protein_L14	-1.298235032	2.11E-05	1.70E-05
SMU_496	putative_cysteine_synthetase_A	-1.29888231	0.000978408	0.000115009
SMU_1288	50S_ribosomal_protein_L19	-1.310925034	0.000188801	0.005222571
SMU_2007	50S_ribosomal_protein_L15	-1.31201405	2.93E-07	7.57E-09
SMU_480	unknown	-1.318244121	6.01E-07	4.29E-07
SMU_1586	threonyl-tRNA_synthetase	-1.322061386	7.12E-07	1.97E-06
SMU_2010	50S_ribosomal_protein_L18	-1.323783862	8.59E-06	0.00015247
SMU_2008	50S_ribosomal_protein_L30	-1.325572739	1.38E-05	7.54E-05
SMU_585	unknown	-1.333446292	2.78E-06	7.99E-06
SMU_1734	acetyl-CoA_carboxylase_subunit_alpha	-1.34041531	5.36E-09	1.39E-11
SMU_830	RgpFc_protein	-1.342495968	4.22E-07	1.91E-06
SMU_1512	phenylalanyl-tRNA_synthetase_subunit_alpha	-1.347195696	6.34E-05	9.81E-11
SMU_1005	glucosyltransferase-SI	-1.357442728	1.49E-09	5.69E-17
SMU_1711	putative_pseudouridylate_synthase_B%2C_large_subunit	-1.360410215	0.00119707	0.000192183
SMU_08	putative_transcription-repair_coupling_factor	-1.367761366	0.001127538	0.000158155
SMU_2074	anaerobic_ribonucleoside_triphosphate_reductase	-1.369233602	2.29E-09	3.39E-10

SMU_1607	putative_exoribonuclease_R	-1.369754396	8.38E-05	7.79E-07
SMU_30	putative_phosphoribosylformylglycinamidine_synthase	-1.381511576	0.000550229	4.66E-05
SMU_37	phosphoribosylaminoimidazolecarboxamide_formyltransferase2FIMP_cyclohydrolase	-1.382549229	0.00182756	0.000521954
SMU_1510	phenylalanyl-tRNA_synthetase_subunit_beta	-1.421755521	1.67E-08	9.12E-09
SMU_1302	putative_surface_adhesin	-1.43130427	4.38E-05	0.000832246
SMU_1472	putative_single-strand_DNA-specific_exonuclease_RecJ	-1.460775985	8.31E-06	2.17E-11
SMU_667	ribonucleotide-diphosphate_reductase_subunit_beta	-1.473577671	2.12E-07	8.31E-14
SMU_1004	glucosyltransferase-I	-1.501027573	1.11E-16	1.40E-36
SMU_1997	putative_ComX1%2C_transcriptional_regulator_of_competence-specific_genes	-1.594934749	9.38E-05	0.000230972
SMU_20	unknown (MreC, putative cell shape determining protein)	-1.610741689	2.99E-05	3.66E-05
SMU_2042	dextranase	-1.661187284	3.37E-19	9.31E-20
SMU_184	putative_ABC_transporter_metal_binding_lipoprotein	1.685501007	8.00E-07	2.95E-08
